# A cost-effective and humidity-tolerant chloride solid electrolyte for lithium batteries

**DOI:** 10.1038/s41467-021-24697-2

**Published:** 2021-07-20

**Authors:** Kai Wang, Qingyong Ren, Zhenqi Gu, Chaomin Duan, Jinzhu Wang, Feng Zhu, Yuanyuan Fu, Jipeng Hao, Jinfeng Zhu, Lunhua He, Chin-Wei Wang, Yingying Lu, Jie Ma, Cheng Ma

**Affiliations:** 1 Division of Nanomaterials & Chemistry, Hefei National Laboratory for Physical Sciences at the Microscale, CAS Key Laboratory of Materials for Energy Conversion, Department of Materials Science and Engineering, University of Science and Technology of China, Hefei, Anhui China; 2 Key Laboratory of Artificial Structures and Quantum Control, School of Physics and Astronomy, Shanghai Jiao Tong University, Shanghai, China; 3 Beijing National Laboratory for Condensed Matter Physics, Institute of Physics, Chinese Academy of Sciences, Beijing, China; 4 Songshan Lake Materials Laboratory, Dongguan, Guangdong China; 5 Spallation Neutron Source Science Center, Dongguan, China; 6 Neutron Group, National Synchrotron Radiation Research Center, Hsinchu, Taiwan; 7 College of Chemical and Biological Engineering, Zhejiang University, Hangzhou, Zhejiang China

**Keywords:** Chemical engineering, Energy, Solid-state chemistry, Batteries, Batteries

## Abstract

Li-ion-conducting chloride solid electrolytes receive considerable attention due to their physicochemical characteristics such as high ionic conductivity, deformability and oxidative stability. However, the raw materials are expensive, and large-scale use of this class of inorganic superionic conductors seems unlikely. Here, a cost-effective chloride solid electrolyte, Li_2_ZrCl_6_, is reported. Its raw materials are several orders of magnitude cheaper than those for the state-of-the-art chloride solid electrolytes, but high ionic conductivity (0.81 mS cm^–1^ at room temperature), deformability, and compatibility with 4V-class cathodes are still simultaneously achieved in Li_2_ZrCl_6_. Moreover, Li_2_ZrCl_6_ demonstrates a humidity tolerance with no sign of moisture uptake or conductivity degradation after exposure to an atmosphere with 5% relative humidity. By combining Li_2_ZrCl_6_ with the Li-In anode and the single-crystal LiNi_0.8_Mn_0.1_Co_0.1_O_2_ cathode, we report a room-temperature all-solid-state cell with a stable specific capacity of about 150 mAh g^–1^ for 200 cycles at 200 mA g^–1^.

## Abstract (web summary)

Stable inorganic solid electrolytes are instrumental in developing high-voltage Li metal batteries. Here, the authors present the synthesis and electrochemical energy storage properties of a cost-effective and humidity-tolerant chloride solid electrolyte.

## Introduction

Identifying an appropriate solid electrolyte is crucial for enabling the safe, energy-dense all-solid-state Li batteries^1–6^. Recently, chloride superionic conductors were raised as an additional class of promising solid electrolytes^7,8^. They are ionically conductive and easily deformable like sulfides, but in the meanwhile are not plagued by the poor oxidative stability of sulfides^9–13^. With Cl being more electronegative than S^14,15^, the oxidation potential of chlorides is generally much higher (comparable to oxides)^16^, leading to an excellent compatibility with 4V-class cathodes^7,8^. The rare combination of these appealing characteristics rapidly attracted intensive research interest^8^.

Nevertheless, for all the chloride solid electrolytes reported so far, the cost of raw materials is still too high to allow for an efficient industrial application. Unlike sulfide and oxide solid electrolytes, the major materials cost of chloride systems does not lie in the raw materials that contribute Li, but in the non-Li-containing ones. Using a method developed by Hart et al.^17^, we calculated and compared the bulk prices of Li-containing compounds needed to synthesize sulfide, oxide, and chloride solid electrolytes (Supplementary Table 1); the laboratory-scale prices used to infer these bulk prices are also specified in Supplementary Table 2. According to such cost analysis, Li_2_S and Li_2_O are both quite expensive; for certain oxide solid electrolytes this cost can be greatly alleviated by using alternate Li-containing raw materials like Li_2_CO_3_, LiNO_3_, LiOH, or their hydrates^18–20^, which could be as cheap as $10.73/kg, whereas the Li_2_S with a high price of $654.18/kg seems irreplaceable for nearly all the sulfide solid electrolytes. In contrast, the price of LiCl is only $5.88/kg, much lower than all the Li-containing raw materials mentioned above. From this perspective, chloride solid electrolytes seem quite promising in terms of cost-effectiveness. Unfortunately, this advantage no longer exists when the non-Li-containing raw materials are taken into account. The present chloride solid electrolyte systems can be divided into two types: Li_3_MCl_6_^7,14,21–26^ and Li_2_M_2/3_Cl_4_^27^ (M is a non-Li element). Elements that had been acting as M in literature are Y^7,14^, Tb-Lu^21^, Sc^25,27^, and In^22,23^, all of which show very low abundance in Earth’s crust, ranging from 0.25 ppm (In) to 33 ppm (Y)^28^. Such values are way below those of the non-Li cationic elements in successfully commercialized solid electrolytes like Li_7_P_3_S_11_^10,11^ (P: 1050 ppm^28^) and Li_1.3_Al_0.3_Ti_1.7_(PO_4_)_3_^29^ (Al: 82,300 ppm, Ti: 5650 ppm^28^). Consequently, as indicated by the bulk prices in Supplementary Table 3 (inferred from the laboratory-scale prices listed in Supplementary Table 4), the costs for most non-Li-containing chlorides needed to synthesize Li_3_MCl_6_ or Li_2_M_2/3_Cl_4_ are way above $1000/kg. Although the corresponding hydrates are often relatively cheap (their bulk prices and the laboratory-scale prices used for estimation are listed in Supplementary Tables 5 and 6, respectively), they cannot be used directly to synthesize any of the reported chloride solid electrolytes other than Li_3_InCl_6_^8^, and dehydrating them before synthesis would likely make the total cost no lower than that of anhydrous chlorides. These expensive non-Li-containing raw materials completely offset the cost-effectiveness of LiCl ($5.88/kg). In order for all-solid-state batteries to be competitive against existing technologies, it has been proposed that the cost of solid electrolytes needs to be lower than $10/m^2^ (note that this includes both raw-material cost and synthesis/processing cost)^30^. If the solid-electrolyte layer is assumed to exhibit a thickness of 50 μm (which is already quite challenging for inorganic systems^31^), even Li_3_YCl_6_, i.e., the solid electrolyte formed by the least expensive chloride in Supplementary Table 3, exhibits a raw-material cost of $23.05/m^2^. This is already more than twice the $10/m^2^ threshold, not to mention the synthesis/processing cost that has not been taken into account here. Therefore, in terms of cost-effectiveness, the present chloride solid electrolytes are far from satisfactory.

This bottleneck arises from the fact that only 3+ cations may act as M in both Li_3_MCl_6_ and Li_2_M_2/3_Cl_4_ due to the charge balance requirement^7,14,21–26^. Although cations with other valences may be introduced through aliovalent doping^32–34^, a considerable fraction of M still has to be 3+ ones. Then, after excluding the trivalent cations not sufficiently stable at 3+ and those too large or too small to fit in the crystal structure, the remaining options are almost confined to the aforementioned expensive elements, i.e., Y, Tb-Lu, Sc, and In. This is why the 3+ valence requirement for M makes it very difficult, if not impossible, to lower the cost of Li_3_MCl_6_ and Li_2_M_2/3_Cl_4_. In fact, beyond the cost issue, such valence requirement is limiting improvement in many other aspects too. For example, soft acids like Sn^4+^ and As^5+^ may result in compounds that are less likely to undergo hydrolysis in humid atmosphere^35–37^ (although this does not necessarily prevent the material from becoming a hydrate with water molecules fitting into the crystal structure). Ge^4+^ and Sn^4+^ with higher electronegativity (2.02 and 1.72, respectively, Allred-Rochow scale^38^) than the aforementioned expensive elements (1.08–1.49) are believed to result in lower electron density on the anions; if other important factors such as the overall crystal structure and anionic species are the same, this effect would supposedly weaken the Coulombic attraction between anions and Li^+^, enabling more facile ionic transport^39,40^. Regardless, the 3+ valence requirement allows none of them to dominate the M site. Unless a Li-M-Cl system with M being a non-trivalent cation emerges, exploration in many directions would be quite difficult; lowering the materials cost is a particularly important and formidable one among them.

**Fig. 1:**
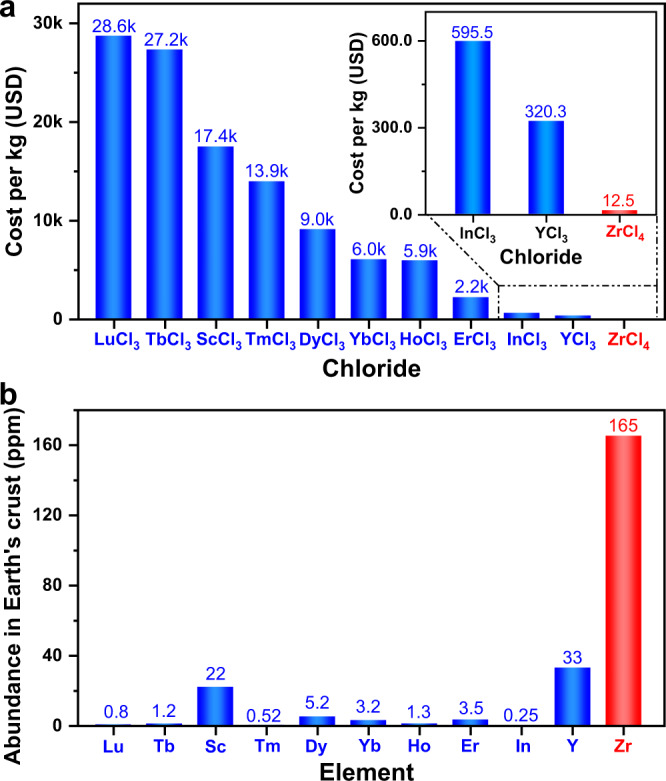
Raw-material cost of Li_2_ZrCl_6_ and the state-of-the-art chloride solid electrolytes. **a** Estimated unit prices of different chlorides when purchased in the quantity of 1000 kg. These chemicals are the raw materials needed to synthesize different chloride solid electrolytes. The bulk prices plotted here are also listed in Supplementary Table 3, and the laboratory-scale prices used to estimate these bulk prices are provided in Supplementary Table 4. **b** Abundance of the corresponding non-Li element in Earth’s crust^28^.

In this work, a cost-effective Li-M-Cl solid electrolyte is successfully constructed using a 4+ cation as M. Described by the chemical formula Li_2_ZrCl_6_, this solid electrolyte is synthesized using ZrCl_4_, which is orders of magnitude cheaper than the raw materials of all the present chloride solid electrolytes (Fig. 1). The raw-material cost of Li_2_ZrCl_6_ at 50 μm thickness is found to be $1.38/m^2^, which is much lower than that for Li_3_YCl_6_ ($23.05/m^2^), i.e., the cheapest chloride system in literature, and is also far below the $10/m^2^ threshold for cost-competitive all-solid-state batteries^30^. With such advantage in cost-effectiveness, Li_2_ZrCl_6_ not only preserves the desirable properties of other chloride solid electrolytes, e.g., high ionic conductivity (0.81 mS cm^–1^), deformability, excellent compatibility with 4V-class cathodes, etc., but also displays a unique stability against moisture. After being exposed to the atmosphere with 5% relative humidity, Li_2_ZrCl_6_ undergoes neither moisture uptake nor conductivity degradation, while Li_3_InCl_6_, i.e., the chloride system considered most humidity tolerant in literature^8,41^, partially becomes Li_3_InCl_6_·2H_2_O and shows significantly decreased conductivity under the same condition.

## Results and discussion

**Fig. 2:**
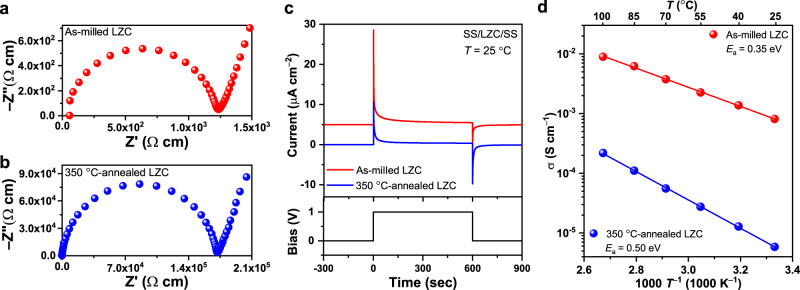
Conductivity of LZC with different processing conditions. **a**, **b** Nyquist plots of the as-milled (**a**) and 350 °C-annealed LZC (**b**) at 25 °C. Note that the data here are not plotted in the unit of resistance, but in the unit of resistivity, i.e., the reciprocal of conductivity, which is calculated using the resistance and sample dimension. The diameters of the pellets used for measurement are 10.8 mm, while the pellet thicknesses are 0.90 and 0.92 mm for the as-milled and 350 °C-annealed LZC, respectively. **c** The transient current behavior under DC bias for the as-milled and 350 °C-annealed LZC with stainless-steel (SS) electrodes. Note that the data of the as-milled LZC here is vertically offset by 5 µA cm^−2^. **d** Arrhenius plots of the as-milled and 350 °C-annealed LZC.

Li_2_ZrCl_6_ (LZC) was mechanochemically synthesized from a stoichiometric mixture of LiCl and ZrCl_4_. At 25 °C, the as-milled LZC was found to exhibit a drastically different ionic conductivity from that annealed at elevated temperatures. As indicated by the electrochemical impedance spectroscopy (EIS) measurement (Fig. 2a), the as-milled LZC shows a high ionic conductivity of 8.08 × 10^–4^ S cm^–1^ at 25 °C. However, annealing at 350 °C for 5 h decreases the ionic conductivity at 25 °C by two orders of magnitude, which reaches 5.81 × 10^–6^ S cm^–1^ as indicated in Fig. 2b (the Bode plots corresponding to these Nyquist plots are shown in Supplementary Fig. 1). With the annealing temperature further increased to 450 °C, the material begins to melt. For both the as-milled and 350 °C-annealed LZC, the electronic conductivities determined by the Hebb–Wagner polarization method^7,42^ (9.22 × 10^–8^ S cm^–1^ and 3.59 × 10^–8^ S cm^–1^ for the as-milled and 350 °C-annealed LZC, respectively, as shown in Fig. 2c) are much lower than the ionic conductivities mentioned above. In particular, the electronic conductivity of the as-milled LZC is about four orders of magnitude lower than its ionic conductivity, which should allow us to safely consider this material as a Li-ion conductor for Li-based batteries. In addition to the room-temperature ionic conductivities, the activation energies were evaluated through the Arrhenius plot (Fig. 2d). Consistent with the measured ionic conductivities, the activation energy of the as-milled LZC (0.35 eV) is much lower than that of the 350 °C-annealed one (0.50 eV), and is comparable with other high-performance chloride solid electrolytes like Li_3_YCl_6_ (0.40 eV^7^) and Li_3_InCl_6_ (0.347 eV^22^). It should be pointed out that all the conductivity measurements described above were performed directly on cold-pressed pellets, and no semicircle, associated with the presence of grain boundaries in the solid electrolyte, is identified in the Nyquist plot of Fig. 2a. This result seems to suggest that the as-milled LZC might be easily deformable and could favor the formation of an intimate electrode-electrolyte contact in all-solid-state cells. In addition to LZC, preliminary attempts were made to explore two similar halides, Li_2_ZrF_6_ and Li_2_ZrBr_6_. The phase-pure Li_2_ZrF_6_ samples can be successfully synthesized using the similar method as that for LZC (Supplementary Fig. 2). However, as shown in Supplementary Fig. 3, both the as-milled and the annealed Li_2_ZrF_6_ exhibit very low ionic conductivities of only 10^−9^−10^−8^ S cm^−1^ and electronic conductivities quite close to these values. Therefore, they do not seem to be ideal solid electrolytes for Li batteries. As for Li_2_ZrBr_6_, we calculated its electrochemical stability using the established scheme^16,43,44^ based on Materials Project^45^, and found that the oxidation potential is only 3.14 V vs. Li/Li^+^ (Supplementary Fig. 4). That is, this bromide, unlike the chloride solid electrolytes, seems unstable against the 4 V-class cathodes. Consequently, the investigation below is still focused on LZC.

**Fig. 3:**
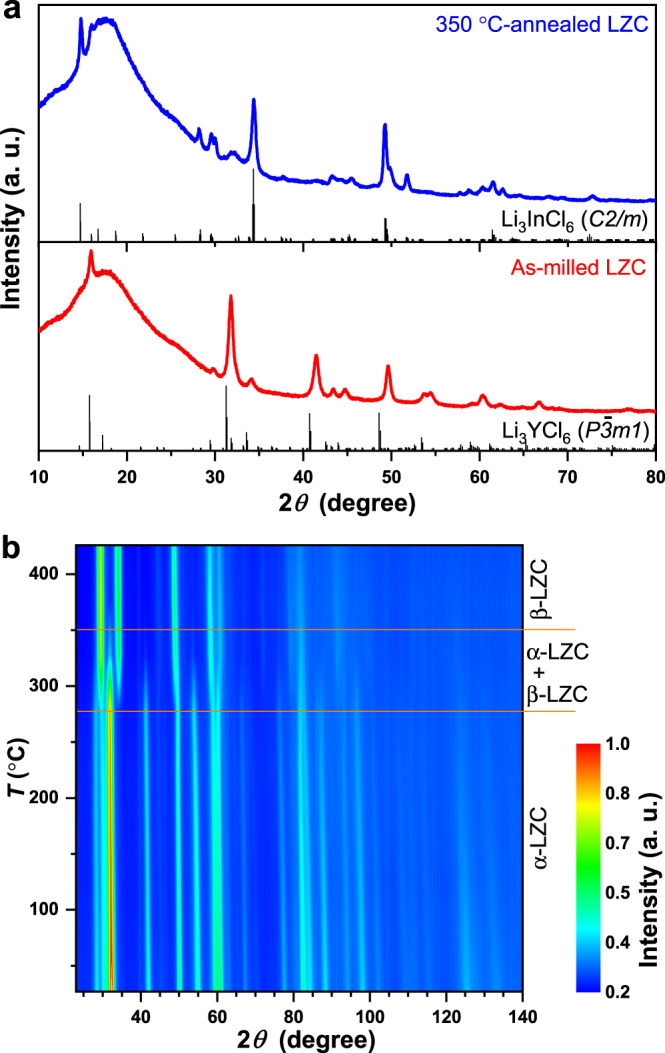
Crystal structures and phase transition of LZC. **a** XRD patterns of the as-milled and 350 °C-annealed LZC. The broad hump below 30° comes from the Kapton film that was used to prevent air exposure. The Bragg positions of Li_3_InCl_6_ and Li_3_YCl_6_ correspond to the structures reported in references 22 and 7, respectively. **b** Two-dimensional intensity color map of temperature-dependent NPD patterns for the as-milled LZC. The data were obtained in-situ between 27 and 427 °C.

While LZC inherits the excellent deformability and ionic conductivity of the state-of-the-art chloride solid electrolytes, the evolution of its crystal structure with processing conditions is quite different from these materials. The X-ray diffraction (XRD) patterns of the as-milled and 350 °C-annealed LZC are displayed in Fig. 3a. The former shows weak and diffuse reflections, suggesting that the intense planetary mill led to a low crystallinity. The Bragg reflections of the as-milled LZC very much resemble those of Li_3_YCl_6_ with the *P*$\overline{3}$*m1* symmetry^7,14^. This phase is referred to as α-LZC below. In contrast, the 350 °C-annealed LZC, besides showing improved crystallinity and sharper reflections, exhibits a completely different crystal structure. The reflections appear to match well with those of Li_3_InCl_6_ crystalizing in the *C2/m* space group^22,23^. This phase is referred to as β-LZC hereafter. Clearly, unlike other chloride solid electrolytes, LZC undergoes changes not only in crystallinity, but also in crystal structures after being annealed at high temperatures.

The phase evolution of LZC was investigated in further detail by in-situ neutron powder diffraction (NPD). The measurement was performed on the as-milled LZC between 27 and 427 °C in argon atmosphere. As shown in Fig. 3b, at first the reflections corresponding to α-LZC persisted with increasing temperature. Starting from 277 °C, the β-LZC reflections (representative ones include 29.4°, 34.1°, 48.7°, and 58°) emerged and grew stronger in the consumption of the α-LZC reflection intensities. The α- and β-LZC phases coexisted between 277 and 350 °C. At higher temperatures, β-LZC is the only phase identified in the NPD pattern. Considering that the 350 °C-annealed (and then furnace-cooled) material still shows pure β-LZC phase (Fig. 3a) instead of becoming α-LZC again, the latter should likely be a meta-stable phase resulting from the intense planetary mill. This is drastically different from other chloride solid electrolytes, where planetary mill may only change the crystallinity, but not the crystal structure^7,15,23^.

**Fig. 4:**
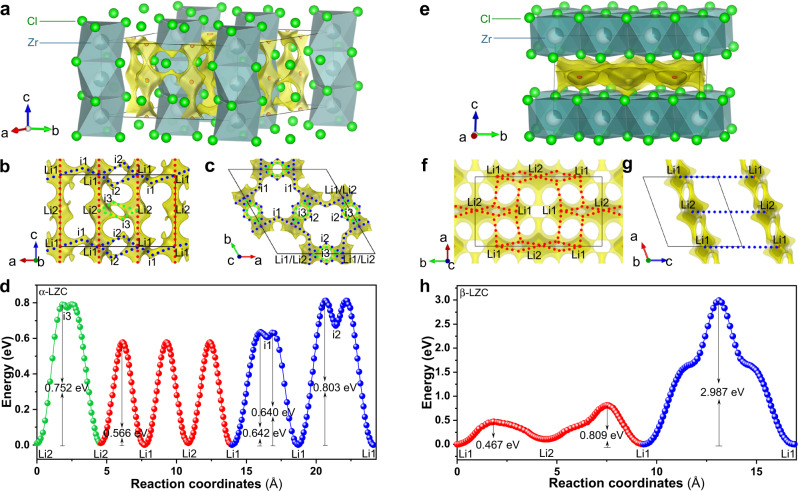
BVSE analysis of Li-ion migration within the α-LZC and β-LZC structures. **a**, **e** The crystal structures of α-LZC (**a**) and β-LZC (**e**) superimposed with the Li-ion potential map. **b**, **c**, **f**, **g** Li-ion migration pathways of α-LZC (**b**,**c**) and β-LZC (**f**, **g**). **d**, **h** Energy profiles of the migration pathways in α-LZC (**d**) and β-LZC (**h**). Each pathway in **b**–**c** and **f**–**g** corresponds to the energy profile of the same color in **d** and **h**, respectively. The crystal structures and potential isosurfaces are visualized using VESTA^56^.

Since the as-milled and 350 °C-annealed LZC differ in not only the crystallinity but also the crystal structure, the contribution of these two factors to the ionic conductivity needs to be discussed separately. To begin with, the influence of crystal structure was investigated through Rietveld refinement. The structure of the as-milled LZC is determined directly from its NPD data. The analysis of the 350 °C-annealed LZC is based on the XRD pattern, but the initial structure used for this refinement is obtained from the NPD pattern of the as-milled sample at 427 °C (Supplementary Fig. 5), which shows the β-LZC structure just like the 350 °C-annealed LZC at room temperature. In this way, only the minor structural variations caused by the different temperatures and processing conditions need to be adjusted when refining the XRD pattern. The initial models used for refining the α- and β-LZC phases are the crystal structures of Li_3_YCl_6_ (space group *P*$$\overline{3}$$*m1*) and that of Li_3_InCl_6_ (space group *C2/m*), respectively, with necessary adjustment of Li contents and the non-Li cation. During the refinement, compositional constraints were applied to ensure that the overall Li:Zr:Cl ratio remains as 2:1:6, while the occupancy of each atomic site was still allowed to vary freely. Such refinement shows excellent agreement between the experimental and calculated data (Supplementary Figs. 5–7), suggesting that α- and β-LZC are indeed structurally similar with Li_3_YCl_6_ and Li_3_InCl_6_, respectively. Using the theoretical density calculated from the refined structure in Supplementary Table 7 (2.561 g cm^–3^) and the prices listed in Supplementary Tables 1 and 3, the raw-material cost of LZC can be estimated as $1.38/m^2^ at 50 μm thickness. This is much more affordable even than the chloride system with the lowest raw-material cost in literature, Li_3_YCl_6_ ($23.05/m^2^), and far below the $10/m^2^ threshold for ensuring the competitiveness of all-solid-state batteries^30^. Beyond such cost-effectiveness, the difference between LZC and the state-of-the-art isostructural chloride systems mainly lies in two aspects. First of all, the lattice parameters of LZC (Supplementary Tables 7 and 8) are generally smaller. This is consistent with the fact that Zr^4+^ is smaller than both Y^3+^ and In^3+^ at the same coordination number^46^. Secondly, the site occupancies are very different too. For example, the 2*a* and 4*g* sites (the Zr ones) in the 350 °C-annealed LZC are much less occupied than those in Li_3_InCl_6_^22^. In addition, while the two Li sites (6*g* and 6*h*) in Li_3_YCl_6_ are both well occupied (occupancy > 0.50)^7^, the Li ions in the as-milled LZC seem to preferentially reside in the 6*h* site. In order to probe the influence of these structural characteristics on ionic transport, we calculated the migration pathways and the associated energy barriers using a bond valence site energy (BVSE) method developed by Adams et al.^47,48^. For the α-LZC structure (schematically shown in Fig. 4a), the [Li1–Li2–Li1] chain along the [001] direction was found to be the most favorable 1D migration pathway (marked in red in Fig. 4b–d). In the meanwhile, Li ions may also migrate along [Li1–i1–Li1-i2-Li1] and [Li2–i3–Li2] (marked in blue and green, respectively, in Fig. 4b–d), which interconnect the [Li1–Li2–Li1] chains to form a 3D percolating network with an effective migration barrier of 0.803 eV (Fig. 4d). For the β-LZC structure (schematically shown in Fig. 4e), the pathways within the a–b plane (marked in red in Fig. 4f–h) are relatively favorable for Li-ion transport, and exhibit an effective barrier of 0.809 eV (Fig. 4h). In comparison, the barrier for migration between two neighboring a–b planes (marked in blue in Fig. 4g, h) are much higher (2.987 eV). It should be emphasized that such results do not necessarily entail a 2D ionic transport. As a matter of fact, the Zr sites hindering the Li-ion migration between two a–b planes are not fully occupied (Supplementary Table 8). In BVSE analysis, their contribution is overly simplified as an average potential that hinders Li-ion migration at each Zr site, no matter whether the particular Zr site is occupied or not. However, in reality the unoccupied ones among these Zr sites could still allow for the local occurrence of Li-ion migration between the neighboring a-b planes. If such migration is too sluggish or too infrequent to make meaningful contribution to the overall ionic transport, the highest barrier of 0.809 eV within the a–b plane should be regarded as the effective migration barrier of the structure^48^. If not, β-LZC would be a 3D ionic conductor, and there might exist more facile percolation pathways than those within the a–b plane, making the effective migration barrier of the entire structure lower than 0.809 eV. As such, the 0.809 eV barrier resulting from BVSE analysis should not be directly considered as the effective migration barrier for β-LZC, but only its highest possible value.

The effective migration barriers obtained this way provide an important clue about the origin for the high ionic conductivity in the as-milled LZC; admittedly, BVSE analysis cannot be expected to precisely repeat the experimentally measured activation energy, so the discussion here focuses only on the calculated migration barriers of different structures. To begin with, it should first be noted that BVSE analysis only describes ionic transport within crystals, but cannot account for contribution from factors beyond the ideal, periodic atomic arrangements, such as defects, surface structure, particle size, strain, amorphous phase, etc. (they are referred to as nonperiodic features below). The 350 °C-annealed LZC is dominated by the highly crystalline phase, so its ionic transport can be better described by BVSE. In contrast, the as-milled LZC contains not only the crystalline phase, but also significant amount of nonperiodic features due to the intense ball milling. According to BVSE analysis, its crystalline part merely allows for a Li-ion migration comparable to or even slower than that in the 350 °C-annealed LZC (the effective migration barrier of the former, 0.803 eV, is close to the highest possible effective migration barrier of the latter, 0.809 eV), and obviously cannot account for the orders of magnitude higher conductivity in the as-milled LZC. As such, the observed fast ionic transport may only arise from the nonperiodic features created by planetary mill. In order to verify this scenario, we annealed the as-milled LZC at 215 °C for 5 h. Such treatment leaves the crystal structure unchanged, but suppresses the metastable nonperiodic features to a great extent, as reflected by the sharper, stronger reflections (Supplementary Fig. 8). With the amount of nonperiodic features reduced this way, the ionic conductivity decreases from 8.08 × 10^–4^ to 3.22 × 10^–5^ S cm^–1^, and the activation energy increases from 0.35 to 0.43 eV (Supplementary Figs. 9 and 10). Therefore, the high ionic conductivity of the as-milled LZC should primarily originate from the noperiodic features induced by the intense ball milling; in fact, similar phenomena had also been observed in other solid electrolytes^7,8,24,49^. It should be pointed out that the results here cannot identify which specific nonperiodic feature is mainly responsible for the high ionic conductivity, because it is very difficult, if not impossible, to separate the influence of each nonperiodic feature on the diffraction patterns. The achievement of this goal demands systematic investigation using advanced characterization methods with ultrahigh spatial resolution, such as aberration-corrected transmission electron microscopy. If the microscopic origin of the facile ionic transport can be precisely identified, an ionic conductivity even higher than that reported here (0.81 mS cm^–1^) might also be realized through a rational optimization.

With the as-milled LZC confirmed to be highly conductive and deformable, its electrochemical stability was evaluated both theoretically and experimentally. Using the established scheme^16,43,44^ based on Materials Project^45^, the electrochemical stability window (ESW) of LZC was calculated to be 1.75–4.25 V vs. Li/Li^+^ (Supplementary Fig. 11); the reductions originate from Zr^4+^ becoming Zr metal in multiple steps, while the oxidation happens through Cl^–^ becoming Cl_2_. The ESW predicted above is verified by the cyclic voltammetry (CV) measurement, which was performed using a Li | Li_7_P_3_S_11_-LZC | LZC + C cell; the LZC + C part in such a cell contains 70 wt% of as-milled LZC and 30 wt% of carbon to make the redox peaks more easily detectable, and the purpose of incorporating a Li_7_P_3_S_11_ layer is to prevent the reaction between LZC and Li. Consistent with the calculated ESW, the CV measurement (Supplementary Fig. 12) reveals multiple reduction peaks below 2 V, along with one oxidation peak above 4 V. These corroborating results convey important information about the electrodes that can be contacted directly with LZC in all-solid-state cells. First of all, the non-zero reduction potential of LZC indicates that this material is unstable against Li reduction. Besides, considering that the reduction products are suggested to contain both the electronic conductor (Zr) and ionic conductor (LiCl), the reaction between LZC and Li metal may not be self-limited, but would likely proceed continuously, similar to the situation for Li_3_YCl_6_ and Li_3_InCl_6_^50,51^. This scenario is supported by the electrochemical measurements carried out in the Li | LZC | Li symmetric cell, whose Li stripping/plating voltages increase constantly without any sign of stabilizing and reach the instrument limit of 5 V in only 88 h (Supplementary Fig. 13a). Besides, LZC would significantly discolor after being contacted directly with Li (Supplementary Fig. 13b−c), further confirming the severe reaction between these two materials. Consequently, like other chloride solid electrolytes^50,51^, LZC should not be in direct contact with Li in all-solid-state cells. Nevertheless, this material does exhibit a rather high oxidation potential beyond 4 V, which could entail a good compatibility with the 4 V-class cathodes. This promising characteristic warrants more detailed investigation of LZC in all-solid-state cells.

**Fig. 5:**
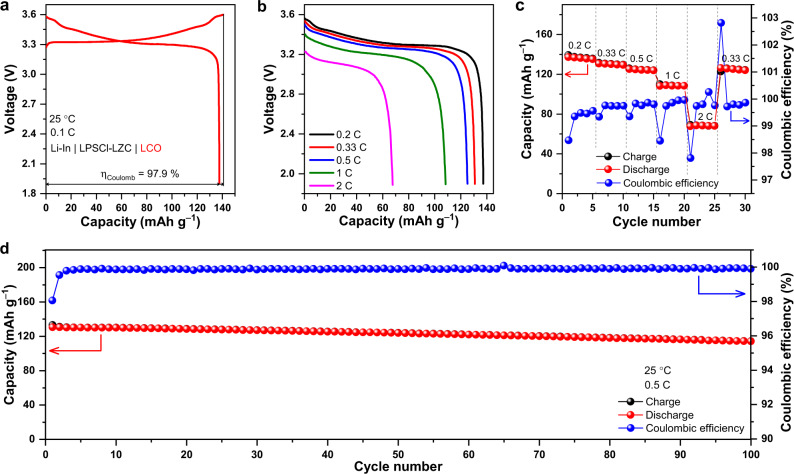
Electrochemical performance of the Li-In | LPSCl-LZC | LCO cell. **a** The initial charge/discharge curves at 0.1 C, with the Coulombic efficiency *η*_Coulomb_ denoted. **b**,**c** Rate capability at 0.2, 0.33, 0.5, 1 and 2 C. **d** Long-term cycling performance at 0.5 C.

**Fig. 6:**
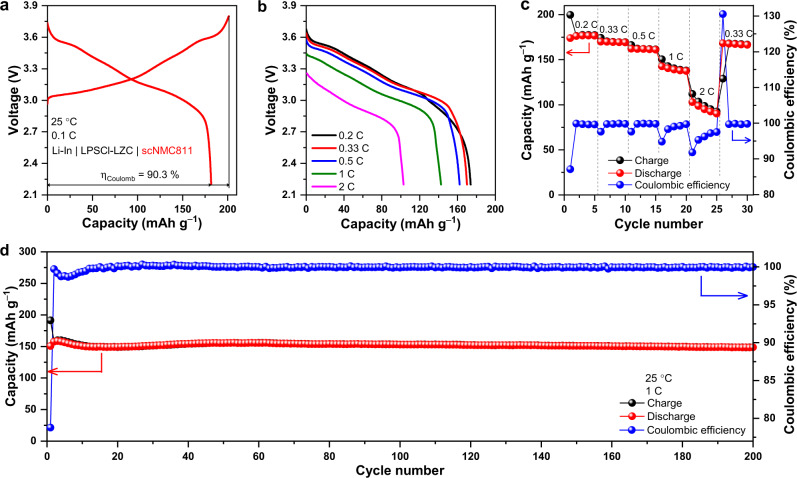
Electrochemical performance of the Li-In | LPSCl-LZC | scNMC811 cell. **a** The initial charge/discharge curves at 0.1 C, with the Coulombic efficiency *η*_Coulomb_ denoted. **b**,**c** Rate capability at 0.2, 0.33, 0.5, 1 and 2 C. **d** Long-term cycling performance at 1 C.

To this end, the as-milled LZC was integrated as the solid electrolyte into all-solid-state cells with LiCoO_2_ (LCO) or single-crystal LiNi_0.8_Mn_0.1_Co_0.1_O_2_ particles (scNMC811) powder as the cathode and Li-In alloy as the anode; to prevent the reaction between the anode and LZC, they are separated by a thin layer of Li_6_PS_5_Cl (abbreviated as LPSCl below), which was applied to the surface of the LZC layer before attaching the anode (further details in Methods). Such a cell was fabricated simply by cold pressing, and bare cathode particles were in direct contact with the as-milled LZC, without any extra coating that is typically needed for sulfide solid electrolytes. When cycled at 0.1 C (1 C = 140 mA g^–1^) between 1.9 and 3.6 V, the cell with LCO cathode (referred to as the LCO/LZC cell below) showed an initial Coulombic efficiency of 97.9% and a discharge capacity of 137 mAh g^–1^ (Fig. 5a), comparable to those delivered by similar cells constructed using Li_3_InCl_6_^23^ (92% and 127 mAh g^–1^) and Li_3_YCl_6_^7^ (94.8% and 118 mAh g^–1^). The rate capability of the LCO/LZC cell is shown in Fig. 5b and c. When the rate increased step-by-step from 0.2 C to 0.5 C, the capacity only dropped slightly; the average capacities at 0.2 C, 0.33 C, and 0.5 C are 136, 130, and 124 mAh g^–1^, respectively. Even at 2 C, a capacity of 68 mAh g^–1^ on average was still retained. Beyond the rate capability, the cycling stability is also examined. The LCO/LZC cell maintained a Coulombic efficiency of 99.9% and a discharge capacity of 114 mAh g^–1^ after 100 cycles at 0.5 C. The ex-situ SEM observation suggests that long-term cycling barely compromised the contact between LCO and LZC (Supplementary Fig. 14), while the energy dispersive spectroscopy (EDS) mapping (Supplementary Fig. 14) and XRD (Supplementary Fig. 15) conducted post-mortem to the composite cathodes after 100 cycles do not indicate any reaction or inter-diffusion happening between the two aforementioned materials either. When LCO was replaced by scNMC811 in the all-solid-state cell (this cell is referred to as the scNMC811/LZC cell below), comparable performances were still observed (Fig. 6). Being cycled at 0.1 C (1 C = 200 mA g^–1^) between 2.2 and 3.8 V, the cell delivered an initial Coulombic efficiency of 90.3% and a discharge capacity of 181 mAh g^–1^ (Fig. 6a). The rate capability of the scNMC811/LZC cell is shown in Fig. 6b and c; the average discharge capacities at 0.2 and 2 C are 176 and 96 mAh g^–1^, respectively. The long-term cycling data is displayed in Fig. 6d, which demonstrates a 99.9% Coulombic efficiency and a 149 mAh g^–1^ discharge capacity after 200 cycles at 1 C. According to the ex-situ SEM (Supplementary Fig. 16) and XRD (Supplementary Fig. 17), the positive electrode and solid electrolyte were still intimately contacted with each other without noticeable reaction or inter-diffusion after prolonged cycling.

In addition to these appealing characteristics, another interesting phenomenon about LZC is its variation with stoichiometry. To study this effect, two off-stoichiometric materials, LiZrCl_5_ and Li_3_ZrCl_7_, were synthesized using the same conditions as the as-milled Li_2_ZrCl_6_. In spite of such large composition variation, these two off-stoichiometric materials remained phase-pure with the α-LZC structure (Supplementary Fig. 18). Besides, their microstructures (Supplementary Figs. 19–21) and calculated ESWs (Supplementary Fig. 22) are also nearly identical with those for the stoichiometric Li_2_ZrCl_6_. However, LiZrCl_5_ and Li_3_ZrCl_7_ exhibit inferior electrochemical properties compared to Li_2_ZrCl_6_. First of all, they are less stable in contact with Li metal electrode; as shown in Supplementary Fig. 23, the Li | LiZrCl_5_ | Li and Li | Li_3_ZrCl_7_ | Li cells reached 5 V Li stripping/plating overpotential in a much shorter time (14.7 h and 48.4 h, respectively) than the Li | Li_2_ZrCl_6_ | Li symmetric cell (88.0 h). Secondly, according to the EIS measurement (Supplementary Fig. 24), the off-stoichiometric materials possess lower ionic conductivity (0.15 and 0.27 mS cm^−1^ for LiZrCl_5_ and Li_3_ZrCl_7_, respectively) than the as-milled Li_2_ZrCl_6_ (0.81 mS cm^−1^). Last but not least, the all-solid-state cell formed by Li_2_ZrCl_6_ (cell configuration Li-In | LPSCl-Li_2_ZrCl_6_ | LCO) deliver higher capacity than those formed by LiZrCl_5_ and Li_3_ZrCl_7_, although all three of them exhibit satisfactory cycling stability (Supplementary Fig. 25). Generally speaking, despite the fact that the material can remain phase-pure in a relatively large stoichiometry range, Li_2_ZrCl_6_ is still the one with the optimal electrochemical performances.

**Fig. 7:**
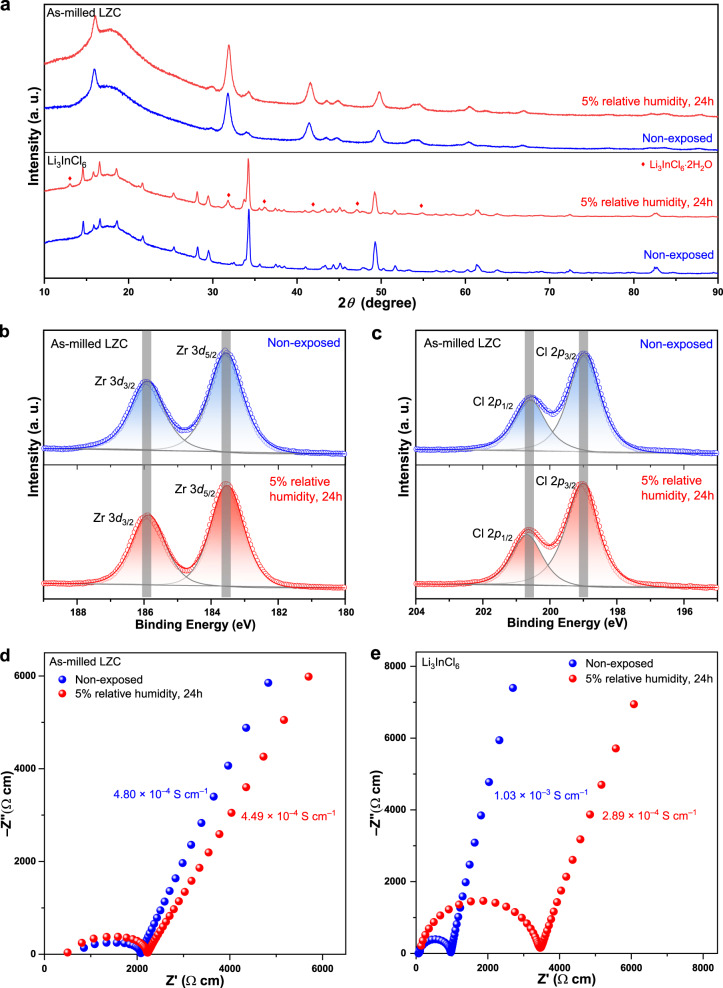
Humidity tolerance of LZC. **a** XRD patterns of the as-milled LZC and Li_3_InCl_6_ before and after being exposed to the atmosphere with 5% relative humidity. **b**, **c** Zr-3*d* (**b**) and Cl-2*p* (**c**) X-ray photoelectron spectra of the as-milled LZC before and after being exposed to the atmosphere with 5% relative humidity. **d**, **e** Nyquist plots of the as-milled LZC (**d**) and Li_3_InCl_6_ (**e**) before and after being exposed to the atmosphere with 5% relative humidity. Note that the data here are not plotted in the unit of resistance, but in the unit of resistivity, i.e., the reciprocal of conductivity, which is calculated using the resistance and sample dimension. The diameters of the pellets used for measurement are all 10.8 mm. The pellet thicknesses are 1.12, 1.10, 1.50, and 1.00 mm for the non-exposed as-milled LZC, humidity-exposed as-milled LZC, non-exposed Li_3_InCl_6_, and humidity-exposed Li_3_InCl_6_, respectively.

Beyond the desirable characteristics shared by most chloride solid electrolytes, LZC also shows a unique advantage in humidity tolerance. The chloride systems reported so far are generally sensitive to moisture. The most humidity tolerant one among them, i.e., Li_3_InCl_6_, will still degenerate even in atmosphere with a very low relative humidity of 1%; its humidity tolerance is in fact a good recoverability after absorbing moisture^23^. In contrast, LZC is truly moisture resistant at relative humidity even above 1%. To demonstrate this point, the as-milled LZC powder was exposed to nitrogen with 5% relative humidity for 24 h. For comparison, Li_3_InCl_6_ powder of the same mass was placed alongside LZC within the same container for the same period of time. According to the XRD patterns (Fig. 7a), the as-milled LZC remained unchanged after such treatment, but Li_3_InCl_6_ partially became Li_3_InCl_6_·2H_2_O. This result is also corroborated by the X-ray photoelectron spectroscopy (XPS) data, where neither the Zr-3*d* nor the Cl-2*p* spectra of the as-milled LZC exhibits any noticeable variation after humidity exposure (Fig. 7b and c). Therefore, the change caused by the moisture, if any, is too trivial to be detected by either XRD or XPS. Consistent with these observations, the as-milled LZC very well preserved its high conductivity after the humidity treatment described above (Fig. 7d), while Li_3_InCl_6_ underwent a conductivity degradation by nearly an order of magnitude (Fig. 7e). The slight difference between the Nyquist plots of LZC before and after its humidity exposure should result from the measurement error of pellet dimensions; with the moisture sensitivity tests performed to loose powders, the EIS measurement before and after humidity exposure cannot be conducted using the same pellet, whereas the conductivities determined using different pellets would inevitably vary slightly due to the measurement error of thicknesses and diameters. Therefore, the conductivity of the as-milled LZC should be considered largely unchanged after humidity exposure. Clearly, in the atmosphere with 5% relative humidity, LZC shows better stability than Li_3_InCl_6_, even though the latter is considered the most humidity tolerant chloride system in literature^8,41^. Since LZC is truly moisture resistant and thus does not need to be recovered after humidity exposure, the environment for its manufacturing and storage does not necessarily possess the low dew point that is typically required by sulfide solid electrolytes. Considering that this material is also quite cost-competitive (Fig. 1), it seems very suitable for large-scale industrial production. The advantages of LZC over state-of-the-art chlorides essentially arise from the fact that a tetravalent cation Zr^4+^, instead of the trivalent ones forming Li_3_MCl_6_ or Li_2_M_2/3_Cl_4_, is acting as the non-Li cation. If 4+ cations other than Zr^4+^ are acting as M in Li_2_MCl_6_, chloride systems showing other interesting properties may very likely arise too.

In summary, Li_2_ZrCl_6_, a cost-effective but high-performance chloride solid electrolyte, is reported. Its raw materials are orders of magnitude cheaper than those for other chloride solid electrolytes, which makes Li_2_ZrCl_6_ presently the only chloride solid electrolyte with raw-material cost ($1.38/m^2^) below $10/m^2^ (the threshold that ensures the competitiveness of all-solid-state batteries^30^). In the meanwhile, the electrochemical performances of the state-of-the-art chloride solid electrolytes are still well preserved. The Li_2_ZrCl_6_ material directly produced by planetary mill shows the *P*$$\overline{3}$$*m1* symmetry, and it undergoes a phase transition into the *C2/m* structure between 277 and 350 °C. Although neither crystal structure seems to favour Li-ion transport according to the BVSE analysis, the as-milled Li_2_ZrCl_6_ was found to possess a desirable room-temperature ionic conductivity of 0.81 mS cm^−1^ due to the nonperiodic features introduced by the intense milling. Besides, this material is easily deformable and compatible with the 4V-class cathode materials. Consequently, without any extra cathode coating that are typically needed for sulfide solid electrolytes, all-solid-state cells with a direct contact between Li_2_ZrCl_6_ and the 4V-class cathode particles can deliver initial Coulombic efficiencies of 97.9% and 90.3% for LiCoO_2_ and scNMC811, respectively. Beyond these common advantages of chloride solid electrolytes, Li_2_ZrCl_6_ also displays humidity tolerance. Instead of being well recoverable after humidity exposure but still moisture sensitive (like Li_3_InCl_6_), Li_2_ZrCl_6_ is truly moisture resistant at 5% relative humidity. Such humidity tolerance, along with the cost-effectiveness, removes two major obstacles to the industrial application of chloride solid electrolytes.

## Methods

### Cost analysis

The prices of the chemicals in bulk purchase were estimated from the laboratory-scale prices using the method proposed by Hart et al.^17^. This method relies on the fact that the unit price, *P*, and the purchase quantity, *Q*, of the chemicals satisfy this general relationship:

1 $$P=a{Q}^{b}$$

where *a* and *b* are constants for a given chemical. If this equation is written in the logarithmic form, it becomes

2 $${{\rm{log }}}_{10}P={{\rm{log }}}_{10}a+b\times {{\rm{log }}}_{10}Q$$

Therefore, based on a series of unit prices *P* and quantities *Q* for the laboratory-scale purchase, the unit price for the purchase in bulk quantity (the amount of 1000 kg was used for the calculations here) can be estimated using Eq. (2) through linear extrapolation, and the reliability of this estimation is reflected by the absolute value of the linear correlation coefficient, |*r* | , between log_10_*P* and log_10_*Q* (|*r* | closer to unity means higher reliability). The laboratory-scale prices used for such estimation are mostly taken from the official website of Alfa Aesar; the only exception is TmCl_3_, whose price is taken from Fisher Scientific. Following the advice by Hart et al.^17^, the laboratory-scale chemicals with the lowest purity were selected for the price estimation. More details about these chemicals, such as the stock number, purchase quantity, and purity, are provided in Supplementary Tables 2, 4 and 6.

### Materials synthesis

Li_2_ZrCl_6_ were synthesized from LiCl (Alfa Aesar, 99.9%) and ZrCl_4_ (Acros Organics BVBA, 98%). The stoichiometric amount of the starting materials were mechanochemically milled in the WC pot using WC balls (5 mm diameter) with a ball-to-powder mass ratio of 10:1. The milling was performed in a planetary mill (FRITSCH, Pulverisette 7 premium line) at 500 rpm for 45 h. Such milling directly yields the as-milled LZC in the main text, while annealing this powder at different temperatures for 5 h leads to other LZC materials discussed in the present study. In order to prevent air exposure, all the annealing experiments were performed with the sample sealed in a vacuum quartz tube. The Li_3_InCl_6_ material used for humidity tolerance test was synthesized by the same method with the annealing temperature of 350 °C; the starting materials were LiCl (Alfa Aesar, 99.9%) and InCl_3_ (Alfa Aesar, 99.99%). The Li_2_ZrF_6_ materials were synthesized by the same method too with the annealing temperature of 500 °C; LiF (Alfa Aesar, 99.98%) and ZrF_4_ (Sigma-Aldrich, 99.9%) were used as the starting materials.

### Structural characterization

The XRD was performed using a Rigaku Ultima IV diffractometer with Cu K*α*1 radiation; the powder was sealed in Kapton film to avoid air exposure. The neutron diffraction study was performed on high intensity neutron diffractometer, WOMBAT, at the OPAL Reactor (Lucas Heights, Australia)^52^ and the General Purpose Powder Diffractometer, GPPD, at the China Spallation Neutron Source. The in-situ NPD experiments were performed at WOMBAT, where around 5 g of the as-milled LZC were loaded in a vanadium can and sealed under an argon atmosphere during measurement. The data were collected at temperatures from 27 to 427 °C with the wavelength of 1.5451 Å. Rietveld refinement was performed using GSAS II^53,54^.

### Conductivity measurements

Prior to the EIS measurement, the powders were cold pressed into pellets at 380 MPa without any heat treatment, and then Au electrodes were sputtered on the pellet surfaces; the sputter coater was placed in the glove box, so that air exposure can be strictly prevented in the entire procedure. The EIS measurement was performed on the cold-pressed pellets acquired above without any external pressure using a MTZ-35 impedance analyzer (Bio-Logic) in the frequency range between 1 Hz and 35 MHz with 10 mV driving potential amplitude. The electronic conductivity was determined by the direct current (DC) polarization measurement on cold-pressed pellets with the applied voltage of 1 V.

### Humidity tolerance test

The humidity tolerance test was conducted by placing 0.5 g as-milled LZC and 0.5 g Li_3_InCl_6_ powders within the same vacuum desiccator filled with N_2_ of 5% relative humidity at 25 °C for 24 h. In order to create this humid environment, we first let dry N_2_ flow through ultrapure water to make it moist. Then, this moisture-containing N_2_ was mixed with dry N_2_ in a certain ratio to reach the desired humidity, which was ensured by a humidity sensor (Shenzhen Everbest Machinery Industry, DT-83, accuracy ±3% relative humidity) placed in the desiccator mentioned above.

### Bond valence site energy (BVSE) calculations

BVSE calculations were performed with the softBV program^47,48^ using structural models obtained from the Rietveld refinement. The energies of different Li sites in the crystal structure were calculated against a 3D grid of points with 0.1 Å resolution using the transferable Morse-type softBV force field. Li-ion migration pathways were identified with the regions of low BVSE.

### Electrochemical characterizations

The ESW of LZC was evaluated by the CV measurement on a Li | Li_7_P_3_S_11_-LZC | LZC+C (weight ratio: LZC/C = 70/30) cell between −0.25 and 5 V at 0.1 mV s^–1^. The cathode composite in the all-solid-state cell for charge/discharge test was prepared by mixing the commercial LiCoO_2_ (Alfa Aesar, 99.5%) or single-cystal LiNi_0.8_Mn_0.1_Co_0.1_O_2_ (Hunan Shanshan Energy Technology, 99.9%) powders with the as-milled LZC (sieved beforehand through 10 μm, 5 μm, and 3 μm meshes) using a vortex mixer (Haimen Kylin-Bell Lab Instruments, QL-866) at 1500 rpm for 10 min in a weight ratio of 75:25. In order to assemble the all-solid-state cell, 60 mg of the as-milled LZC powder was first placed into a polyetheretherketone (PEEK) mold with 10 mm diameter and pressed at 1 ton to form the solid electrolyte layer. Then, 7 mg of the cathode composite powder was dispersed evenly on one side of this solid electrolyte layer and pressed again at 1.2 tons. To avoid reaction between LZC and the anode, a thin layer of Li_6_PS_5_Cl was applied at the other side of the LZC layer in a similar manner: 35 mg of Li_6_PS_5_Cl powder (Shenzhen Kejing Star Technology, 99%) was dispersed evenly on the solid electrolyte layer, and then pressed at 1.2 tons. Afterwards, a piece of indium foil (0.1 mm thick, 10 mm diameter) was placed at the surface of the Li_6_PS_5_Cl layer, and a piece of lithium foil (0.03 mm thick, 10 mm diameter) was subsequently attached to this indium foil. They were then pressed at 1.5 tons to form the Li-In alloy anode^7,55^. The cells were cycled under an external pressure of ~1.5 tons at 25 °C using a LAND CT2001A battery testing system within the voltage ranges of 1.9–3.6 V and 2.2–3.8 V for LiCoO_2_ and scNMC811, respectively; the temperature of 25 °C was ensured by placing the cells in an incubator (Tianjin Hongnuo Instrument, SPX-250B, temperature accuracy ±1 °C) during cycling.

## Supplementary information

Supplementary Information

## Data Availability

The data that support the findings of this study are available within the article (and its Supplementary Information files) and from the corresponding author upon reasonable request.
