# Supplementary material for: A cost-effective and humidity-tolerant chloride solid electrolyte for lithium batteries
Source: Nat Commun. 2021 Jul 20;12:4410. doi: 10.1038/s41467-021-24697-2 (PMC8292426; doi:10.1038/s41467-021-24697-2)
Supplement: Supplementary file 1 — Supplementary Information [file 41467_2021_24697_MOESM1_ESM.pdf]

Supplementary Information

A cost-effective and humidity-tolerant chloride solid electrolyte for lithium batteries

Kai Wang[1], Qingyong Ren[2], Zhenqi Gu[1], Chaomin Duan[1], Jinzhu Wang[1], Feng Zhu[1], Yuanyuan Fu[1], Jipeng Hao[1], Jinfeng Zhu[2], Lunhua He[3,4,5], Chin-Wei Wang[6], Yingying Lu[7], Jie Ma[2], and Cheng Ma[1]*

[1]Division of Nanomaterials & Chemistry, Hefei National Laboratory for Physical Sciences at the Microscale, CAS Key Laboratory of Materials for Energy Conversion, Department of Materials Science and Engineering, University of Science and Technology of China, Hefei, Anhui 230026, China.

[2]Key Laboratory of Artificial Structures and Quantum Control, School of Physics and Astronomy, Shanghai Jiao Tong University, 800 Dongchuan Road, Shanghai 200240, China.

[3]Beijing National Laboratory for Condensed Matter Physics, Institute of Physics, Chinese Academy of Sciences, Beijing 100190, China.

[4]Songshan Lake Materials Laboratory, Dongguan, Guangdong 523508, China.

[5]Spallation Neutron Source Science Center, Dongguan 523503, China.

[6]Neutron Group, National Synchrotron Radiation Research Center, Hsinchu 30077, Taiwan.

[7]College of Chemical and Biological Engineering, Zhejiang University, Hangzhou, Zhejiang 310027, China.

*Corresponding author: mach16@ustc.edu.cn

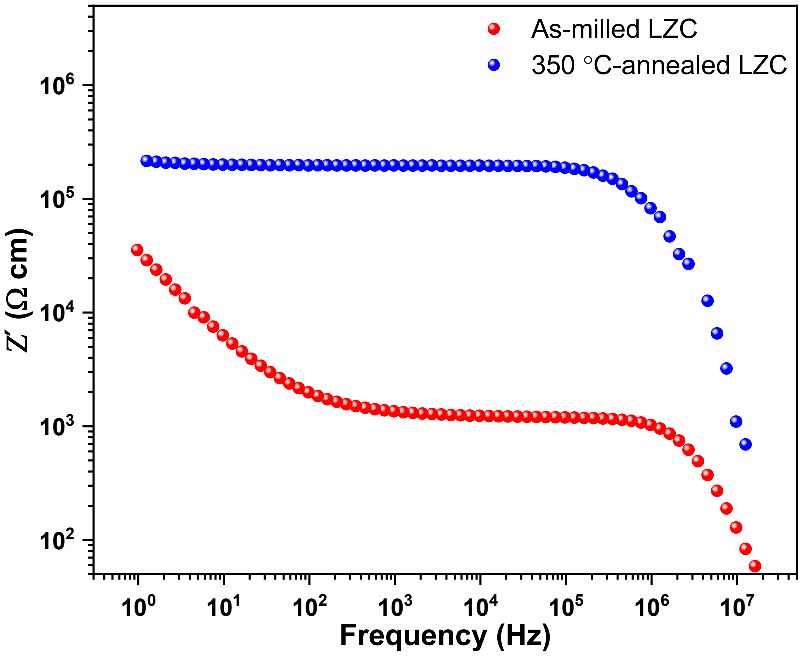

Supplementary Fig. 1. Bode plots corresponding to the Nyquist plots of the as-milled and 350 °C-annealed LZC shown in Fig. 2a–b of the main text. The measurement was conucted at 25 °C.

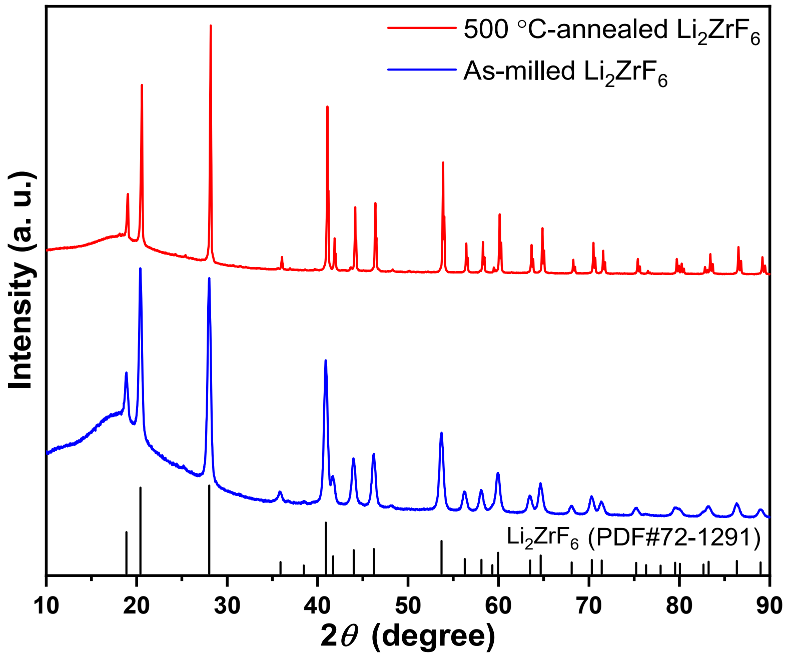

Supplementary Fig. 2. XRD patterns of the as-milled and 500 °C-annealed Li_2ZrF_6.

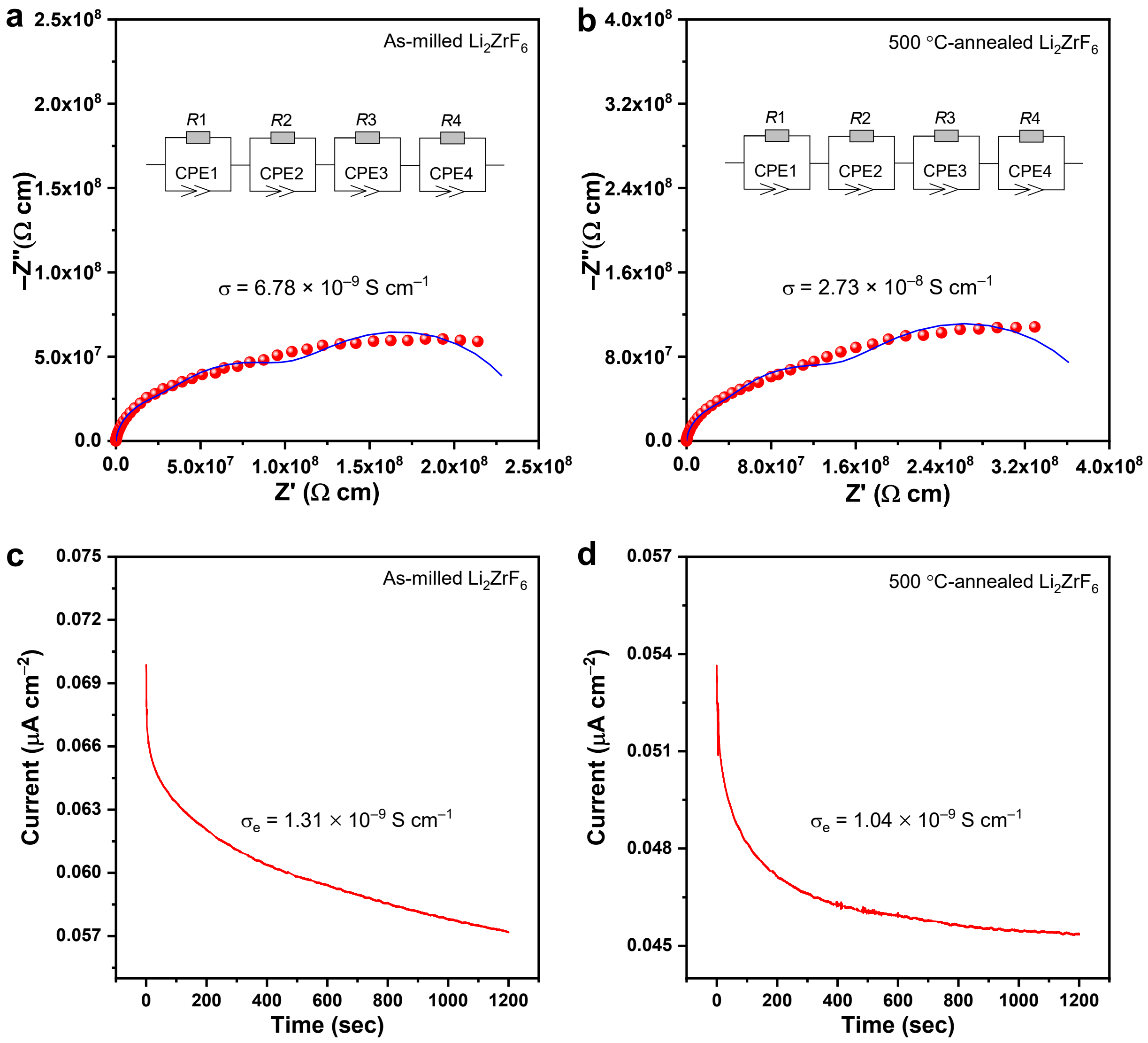

Supplementary Fig. 3. a,**b**, Nyquist plots of the as-milled (**a**) and 500 °C-annealed Li_2ZrF_6 (**b**) at 25 °C. The measurement was performed in the frequency range between 1 Hz and 35 MHz with 5 V driving potential amplitude. Note that the data here are not plotted in the unit of resistance, but in the unit of resistivity, i.e., the reciprocal of conductivity, which is calculated using the resistance and sample dimension. The diameters of the pellets used for measurement are 10.8 mm, while the pellet thicknesses are 1.01 and 1.05 mm for the as-milled and 500 °C-annealed Li_2ZrF_6, respectively. The resistivity values were obtained by fitting the experimental data with the equivalent circuits shown in the insets (*R* stands for resistance and CPE for constant-phase element); the red dots and blue lines are the experimental data and fitted curves, respectively. Since the semicircles in both Nyquist plots

are too large to be covered completely by the frequency range of our instrument (1 Hz – 35 MHz), the tails at frequencies lower than the semicircles are not identifiable, making it impossible to know whether ionic or electronic conductivity is dominant. Therefore, the ionic conductivities may only be estimated, empirically, based on the fitted values of $R1$. **c**,**d**, The transient current behavior under DC bias for the as-milled (**c**) and 500 °C-annealed Li_2ZrF_6 (**d**) with stainless-steel (SS) electrodes. The applied voltage is 5 V, and the measurements were conducted at 25 °C.

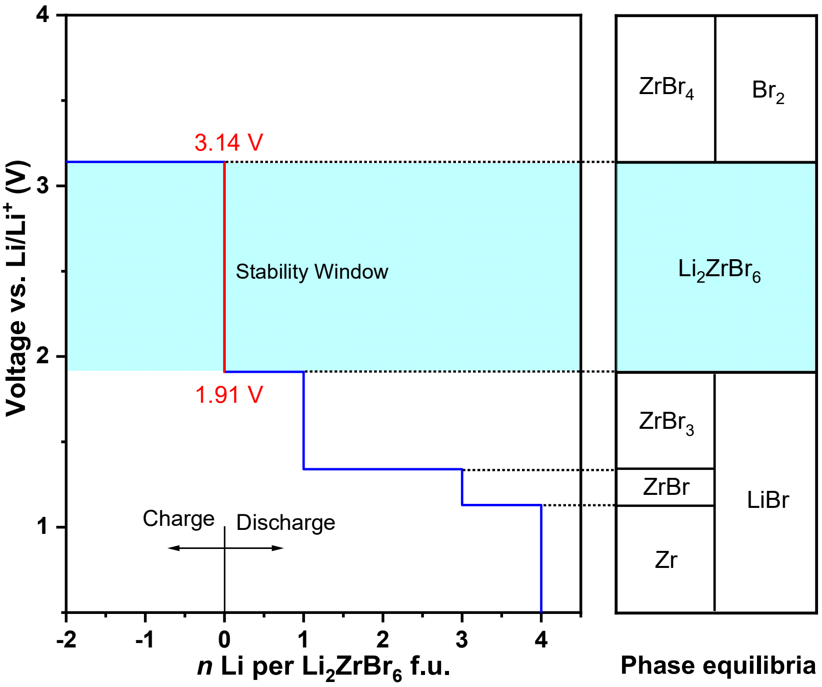

Supplementary Fig. 4. Calculated thermodynamic equilibrium voltage profiles and the phase equilibria for Li_2ZrBr_6.

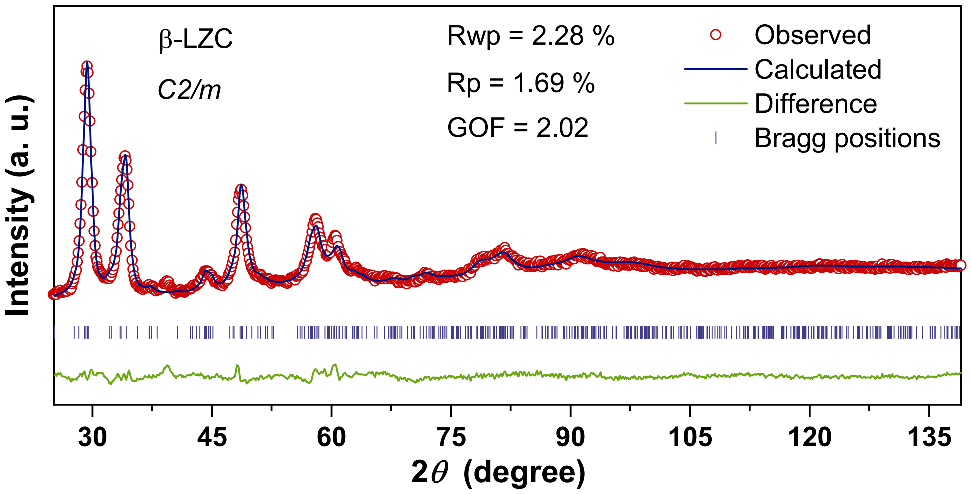

Supplementary Fig. 5. Rietveld refinement of the NPD data for the as-milled LZC at 427 °C.

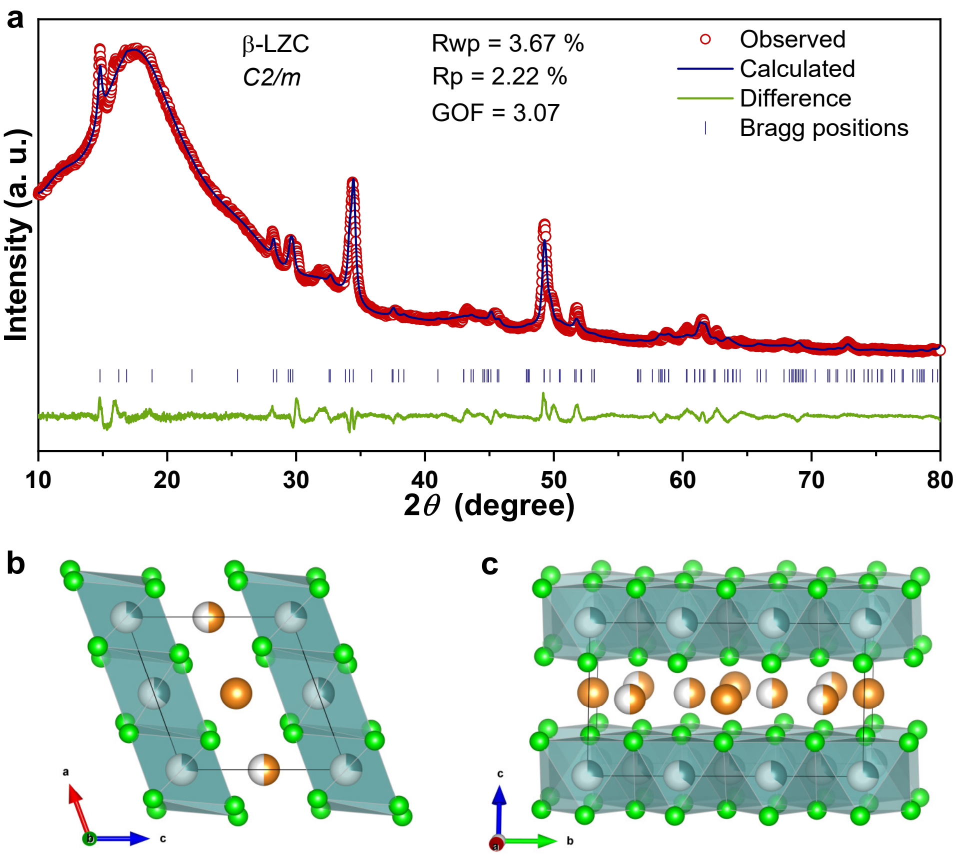

Supplementary Fig. 6. a, Rietveld refinement of the room-temperature XRD data for the 350 °C-annealed LZC. **b**,**c**, The structural model obtained from the Rietveld refinement in **a**. The brown, bluish-grey, and light-green balls represent Li, Zr, and Cl atoms, respectively.

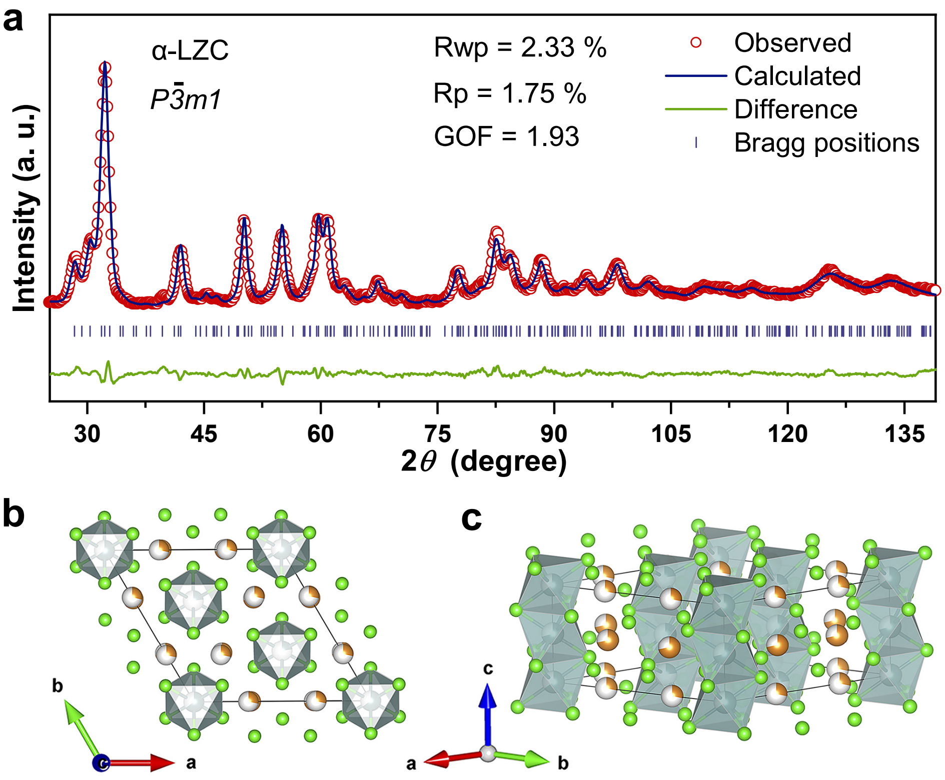

Supplementary Fig. 7. a, Rietveld refinement of the NPD data for the as-milled LZC at 27 °C. **b**,**c**, The structural model obtained from the Rietveld refinement in **a**. The brown, bluish-grey, light-green balls represent Li, Zr, Cl atoms, respectively.

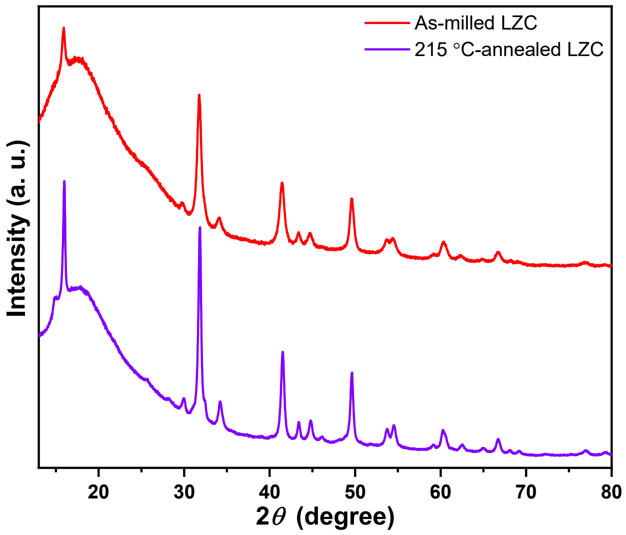

Supplementary Fig. 8. XRD patterns of the as-milled and 215 °C-annealed LZC.

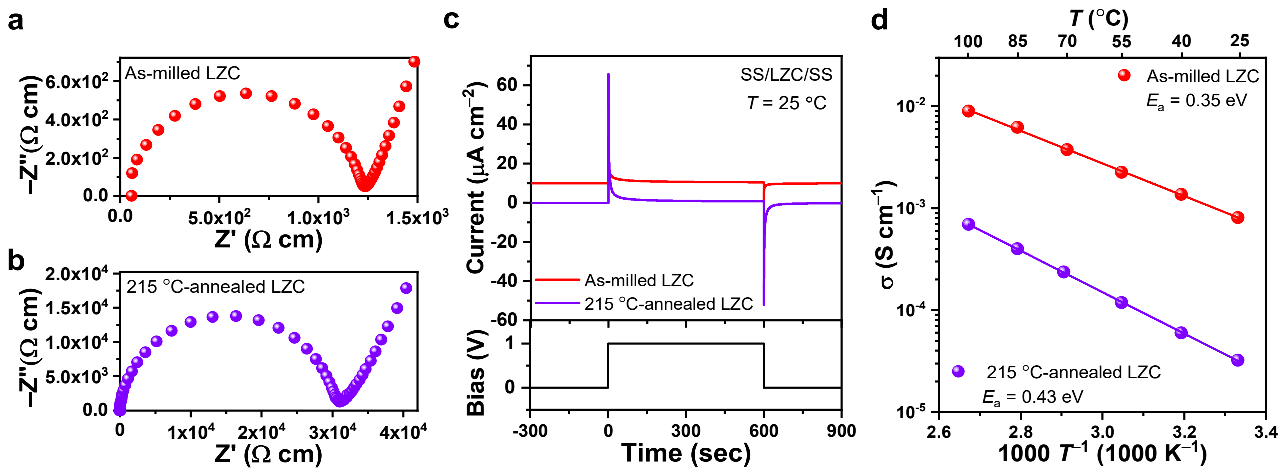

Supplementary Fig. 9. a,**b**, The Nyquist plots of the as-milled (**a**) and 215 °C-annealed LZC (**b**) at 25 °C. Note that the data here are not plotted in the unit of resistance, but in the unit of resistivity, i.e., the reciprocal of conductivity, which is calculated using the resistance and sample dimension. The diameters of the pellets used for measurement are 10.8 mm, while the pellet thicknesses are 0.90 and 1.02 mm for the as-milled and 215 °C-annealed LZC, respectively. **c**, The transient current behavior under DC bias for the as-milled and 215 °C-annealed LZC with stainless-steel (SS) electrodes. The electronic conductivity of the 215 °C-annealed LZC is 7.33×10^{-8} S cm^{-1}. Note that the data of the as-milled LZC is vertically offset by 10 μA cm^{-2}. **d**, Arrhenius plots of the as-milled and 215 °C-annealed LZC.

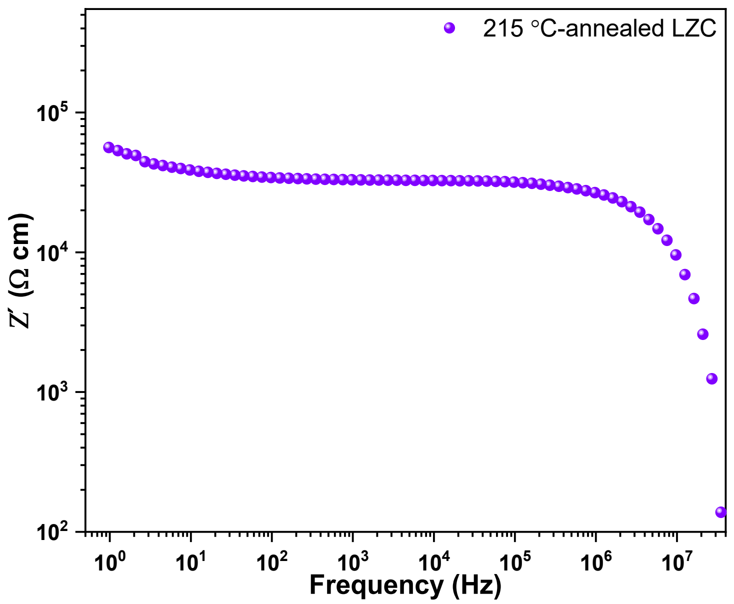

Supplementary Fig. 10. Bode plot corresponding to the Nyquist plot of the 215 °C-annealed LZC shown in Supplementary Fig. 9b. The measurement was conducted at 25 °C.

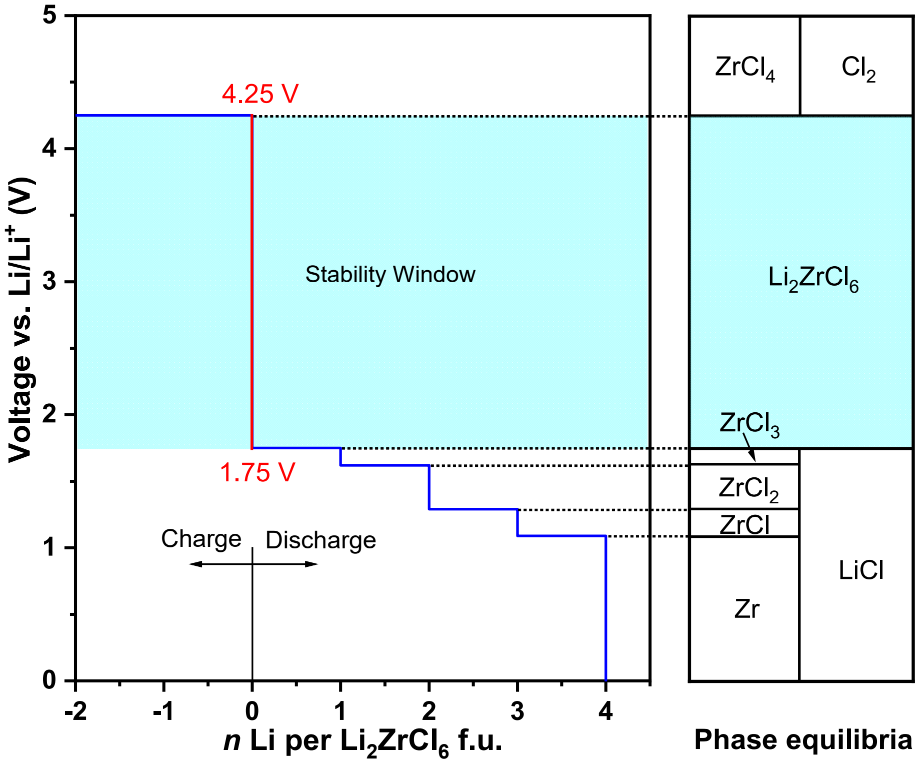

Supplementary Fig. 11. Calculated thermodynamic equilibrium voltage profiles and the phase equilibria for Li_2ZrCl_6.

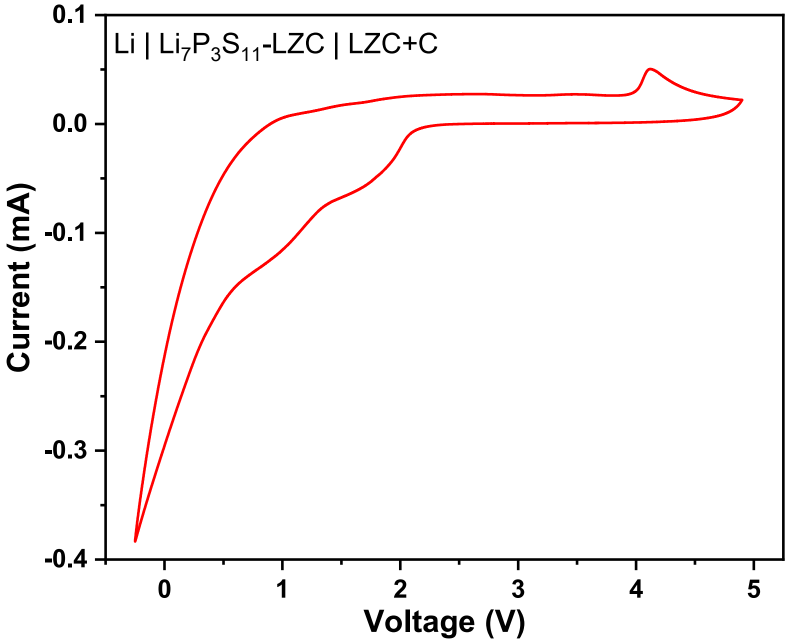

Supplementary Fig. 12. CV curve of the Li | $Li_7P_3S_{11}$-LZC | LZC+C cell at 0.1 mV s^{-1}. The measurement was conducted at 25 °C.

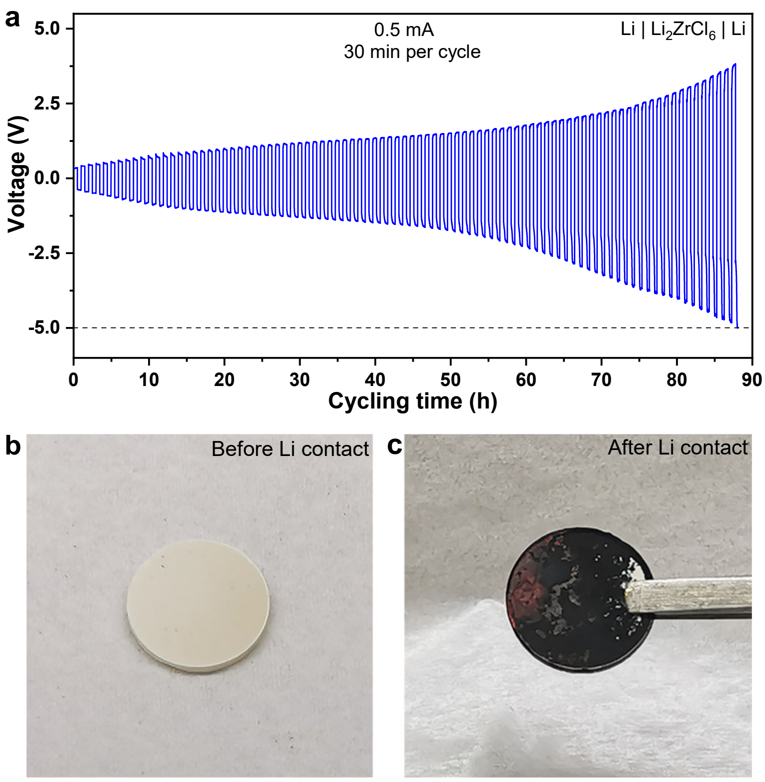

Supplementary Fig. 13. a, Galvanostatic cycling profiles of the symmetric Li | as-milled LZC | Li cell at 0.5 mA (30 minutes per plating/stripping cycle). The measurement was conducted at 25 °C. **b**,**c**, Photographs of the as-milled LZC before (**b**) and after (**c**) contact with lithium for 240 h.

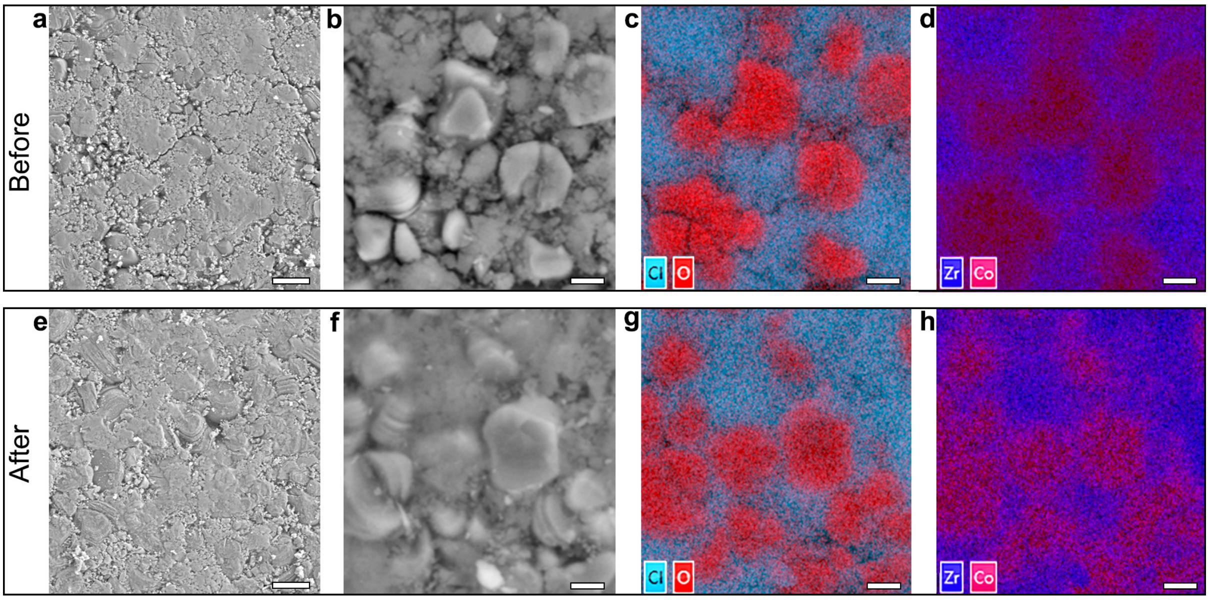

Supplementary Fig. 14. a–**d**, SEM images (**a**,**b**) and EDS mapping results (**c**,**d**) of the composite cathode for the LCO/LZC cell before cycling. **e**–**h**, SEM images (**e**,**f**) and EDS mapping results (**g**,**h**) of the composite cathode for the LCO/LZC cell after 100 cycles at 0.5 C. The scale bars in **a** and **e** are both 5 μm. The scale bars in **b**–**d** and **f**–**h** are 2 μm.

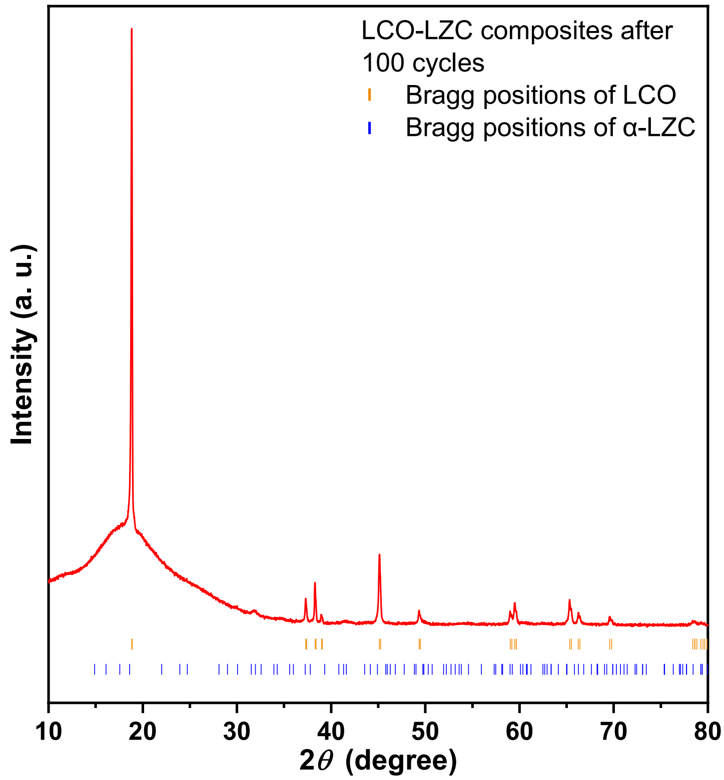

Supplementary Fig. 15. XRD pattern of the composite cathode for the LCO/LZC cell after 100 cycles at 0.5 C.

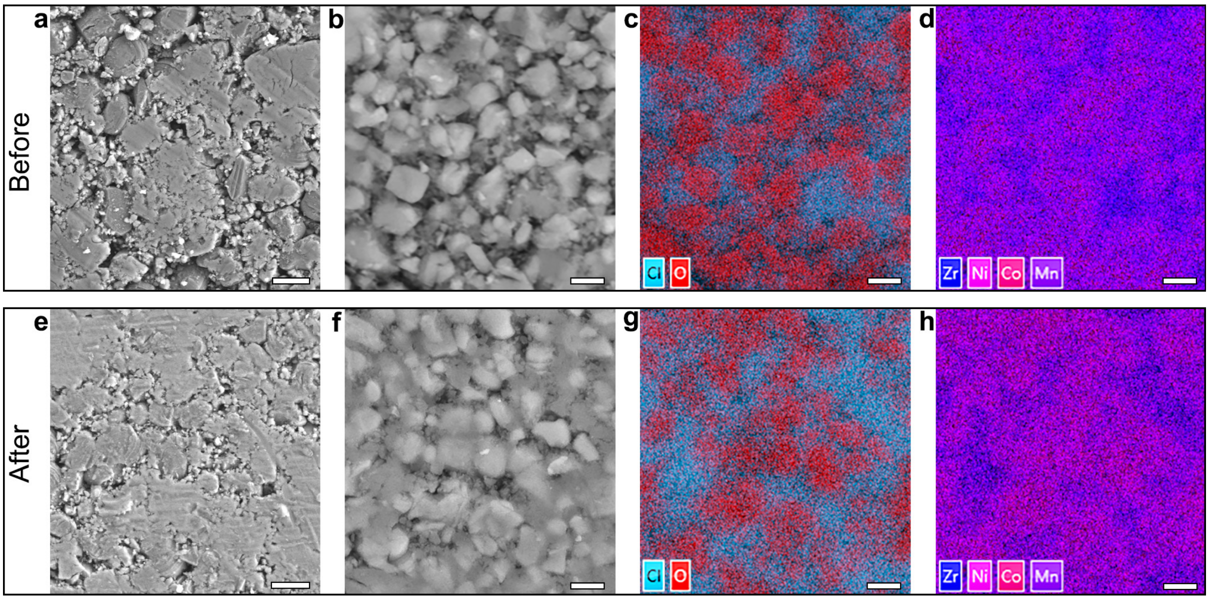

Supplementary Fig. 16. a–**d**, SEM images (**a**,**b**) and EDS mapping results (**c**,**d**) of the composite cathode for the scNMC811/LZC cell before cycling. **e**–**h**, SEM images (**e**,**f**) and EDS mapping results (**g**,**h**) of the composite cathode for the scNMC811/LZC cell after 200 cycles at 1 C. The scale bars are all 2 μm.

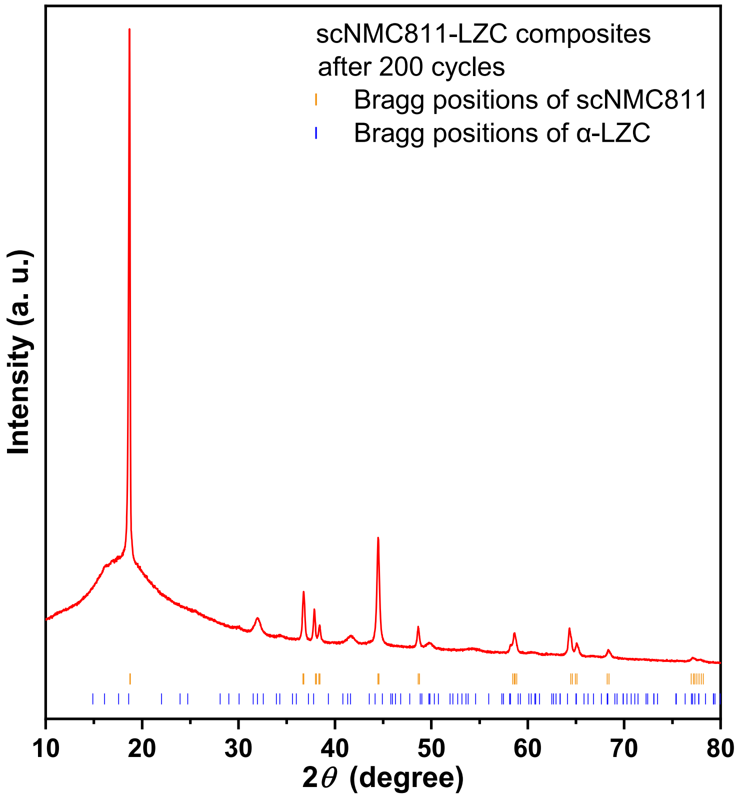

Supplementary Fig. 17. XRD pattern of the composite cathode for the scNMC811/LZC cell after 200 cycles at 1 C.

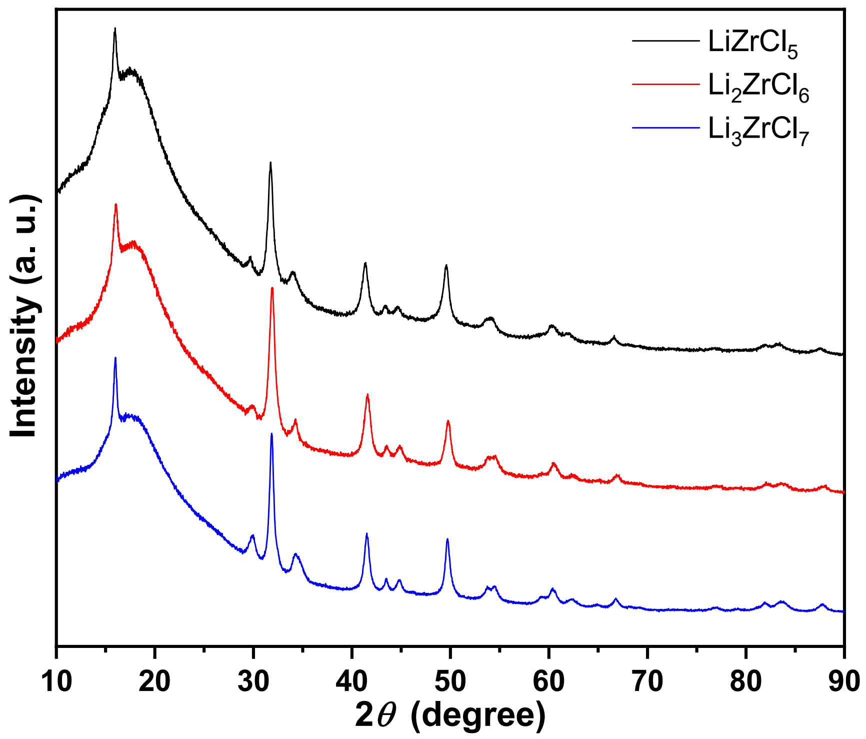

Supplementary Fig. 18. XRD patterns of the as-milled $LiZrCl_5$, Li_2ZrCl_6 and Li_3ZrCl_7.

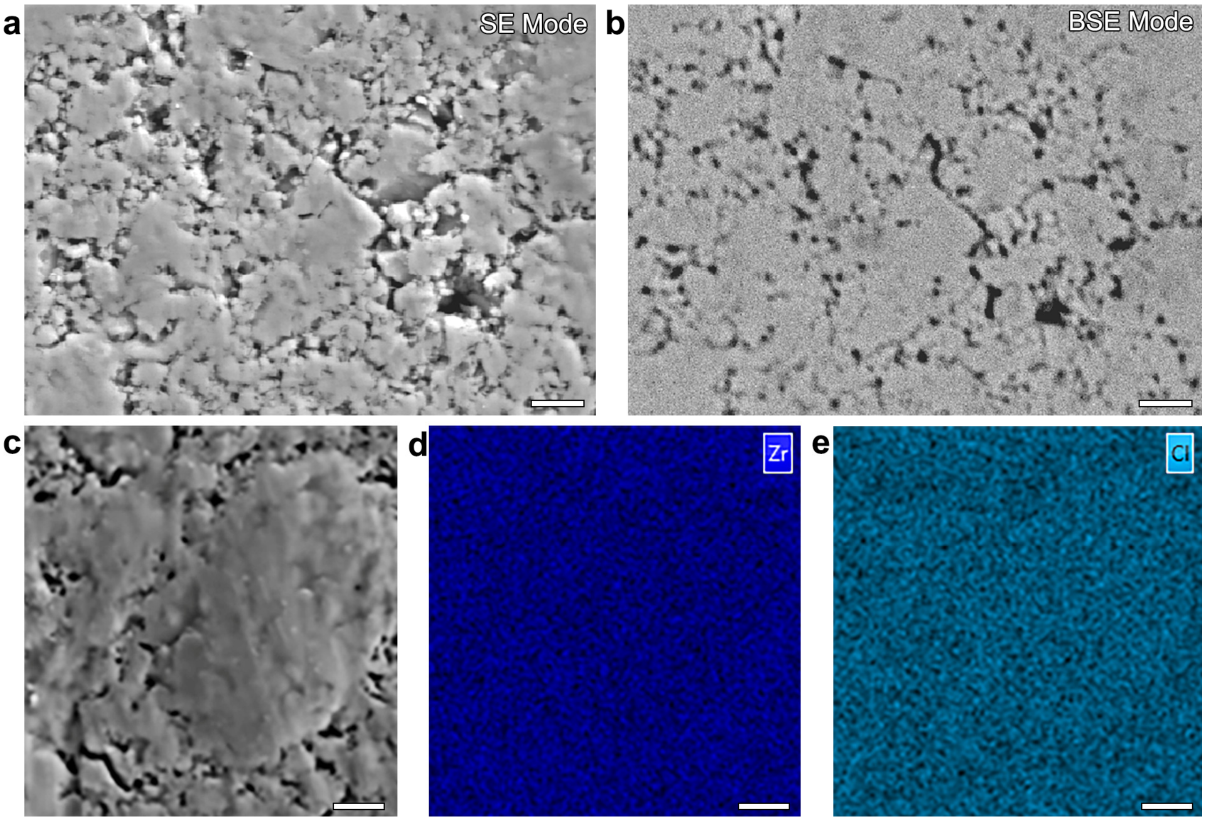

Supplementary Fig. 19. a,**b**, SEM images of the as-milled $LiZrCl_5$ at the secondary-electron (SE) mode (**a**) and backscattered-electron (BSE) mode (**b**). **c**–**e**, secondary-electron SEM image (**c**) and the corresponding EDS mapping results (**d** and **e**) of the as-milled $LiZrCl_5$. The scale bars are all 1 μm.

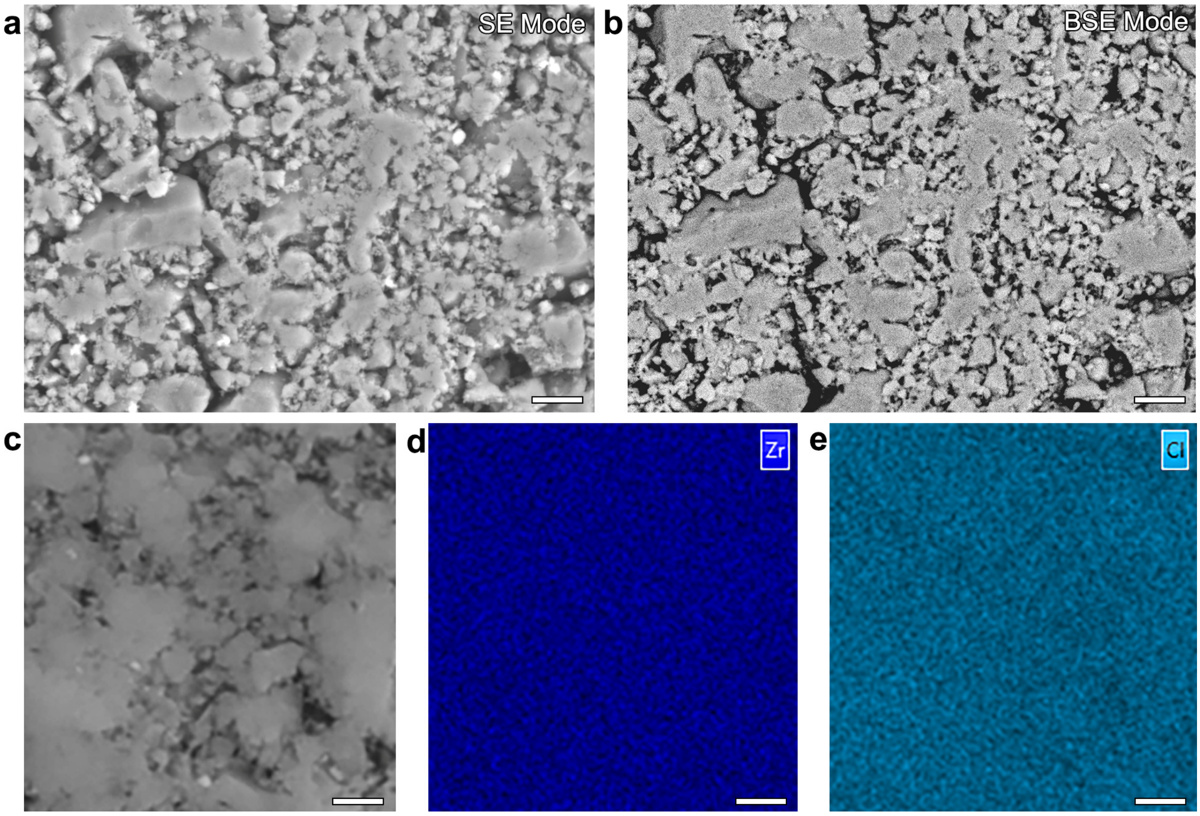

Supplementary Fig. 20. a,**b**, SEM images of the as-milled Li_2ZrCl_6 at the secondary-electron (SE) mode (**a**) and backscattered-electron (BSE) mode (**b**). **c**–**e**, secondary-electron SEM image (**c**) and the corresponding EDS mapping results (**d** and **e**) of the as-milled Li_2ZrCl_6. The scale bars are all 1 µm.

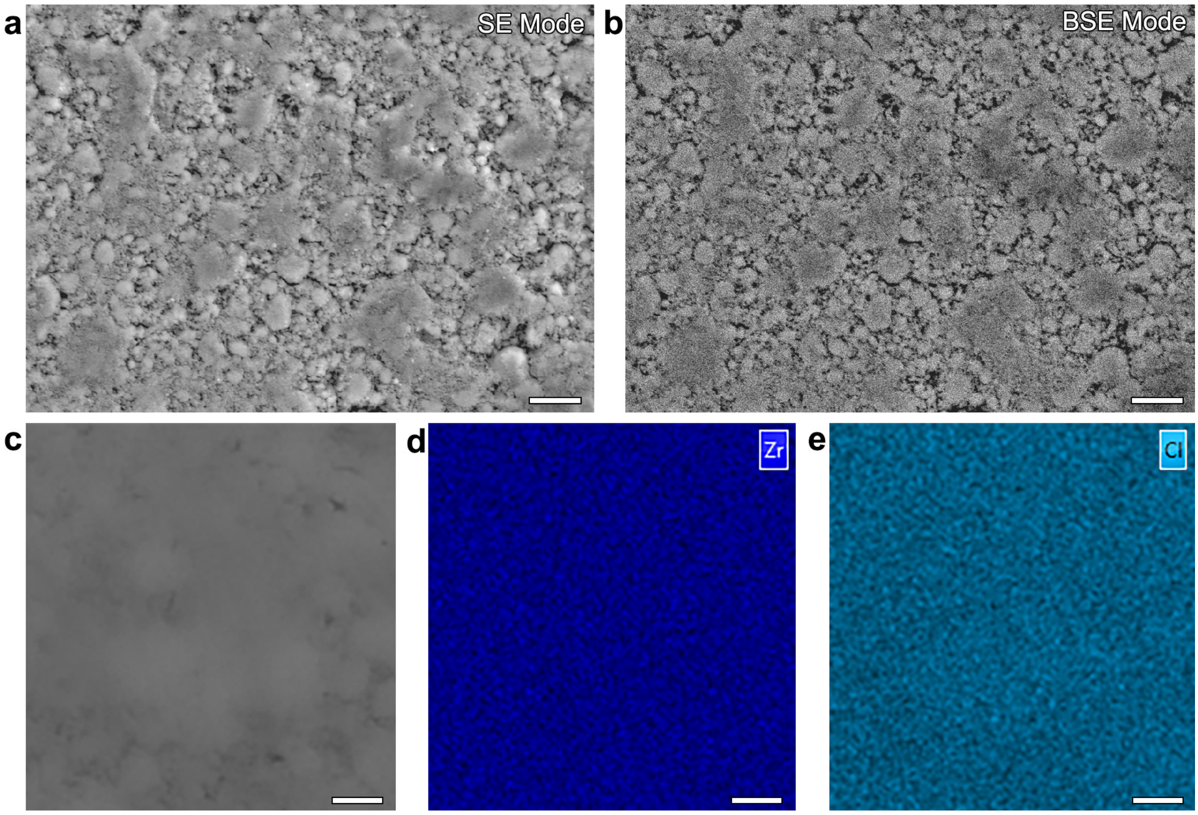

Supplementary Fig. 21. a,**b**, SEM images of the as-milled Li_3ZrCl_7 at the secondary-electron (SE) mode (**a**) and backscattered-electron (BSE) mode (**b**). **c**–**e**, secondary-electron SEM image (**c**) and the corresponding EDS mapping results (**d** and **e**) of the as-milled Li_3ZrCl_7. The scale bars are all 1 µm.

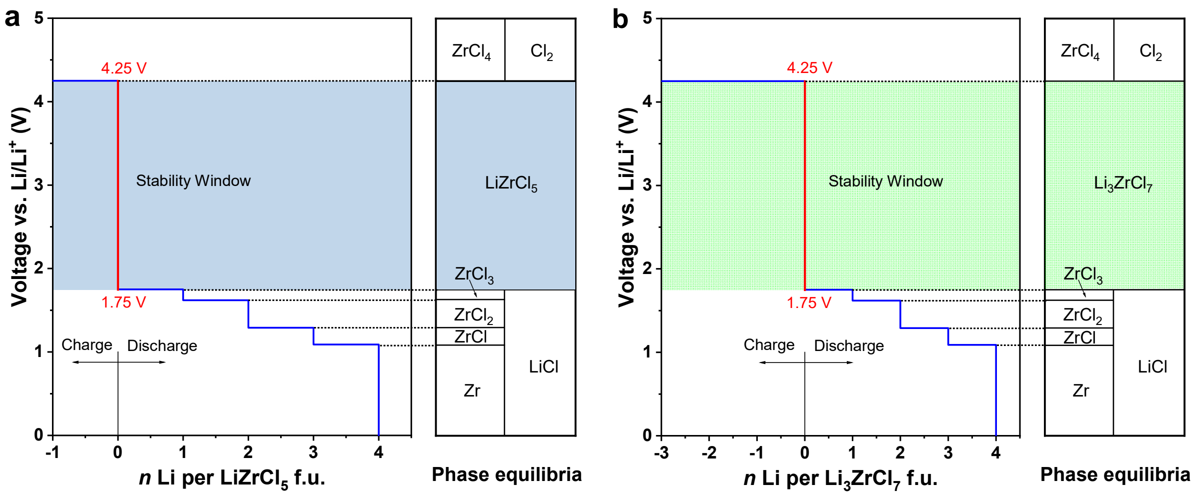

Supplementary Fig. 22. a,**b**, Calculated thermodynamic equilibrium voltage profiles and the phase equilibria for $LiZrCl_5$ (**a**) and Li_3ZrCl_7 (**b**).

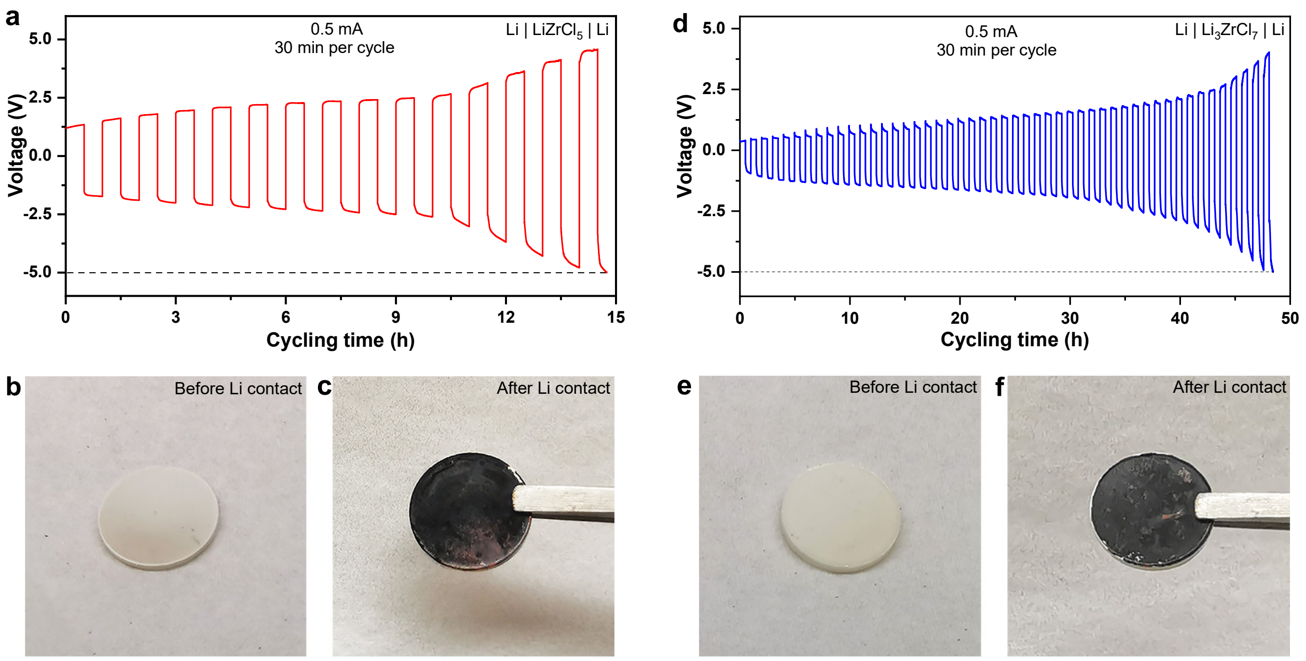

Supplementary Fig. 23. a, Galvanostatic cycling profiles of the symmetric Li | $LiZrCl_5$ | Li cell at 0.5 mA (30 minutes per plating/stripping cycle). The measurement was conducted at 25 °C. **b**,**c**, Photographs of $LiZrCl_5$ before (**b**) and after (**c**) contact with lithium for 240 h. **d**, Galvanostatic cycling profiles of the symmetric Li | Li_3ZrCl_7 | Li cell at 0.5 mA (30 minutes per plating/stripping cycle). The measurement was conducted at 25 °C. **e**,**f**, Photographs of Li_3ZrCl_7 before (**e**) and after (**f**) contact with lithium for 240 h.

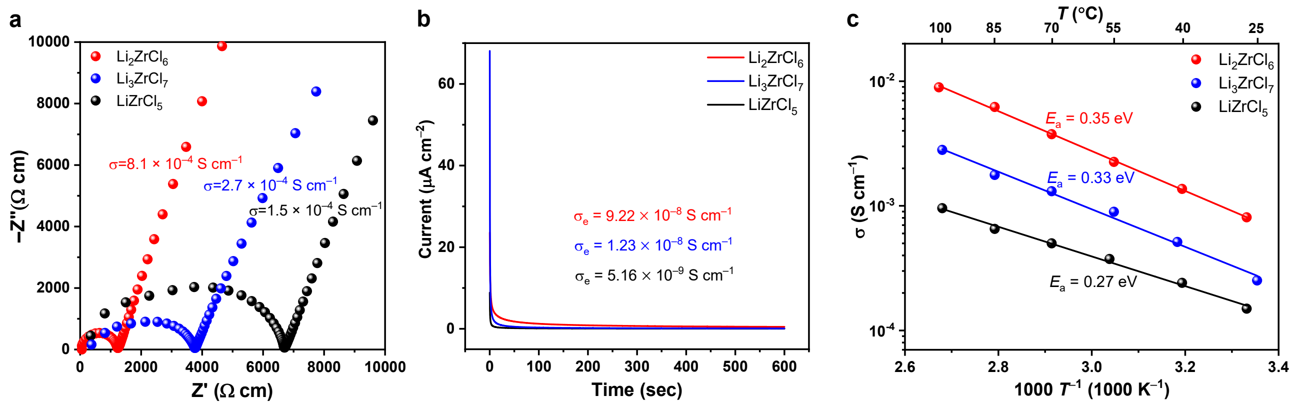

Supplementary Fig. 24. **a**, Nyquist plots of the as-milled $LiZrCl_5$, Li_2ZrCl_6 and Li_3ZrCl_7 at 25 °C. Note that the data here are not plotted in the unit of resistance, but in the unit of resistivity, i.e., the reciprocal of conductivity, which is calculated using the resistance and sample dimension. The diameters of the pellets used for measurement are 10.8 mm, while the pellet thicknesses are 1.20, 0.90, and 1.21 mm for the as-milled $LiZrCl_5$, Li_2ZrCl_6 and Li_3ZrCl_7, respectively. **b**, The transient current behavior under DC bias for the as-milled $LiZrCl_5$, Li_2ZrCl_6 and Li_3ZrCl_7 at 25 °C. **c**, Arrhenius plots of the as-milled $LiZrCl_5$, Li_2ZrCl_6 and Li_3ZrCl_7.

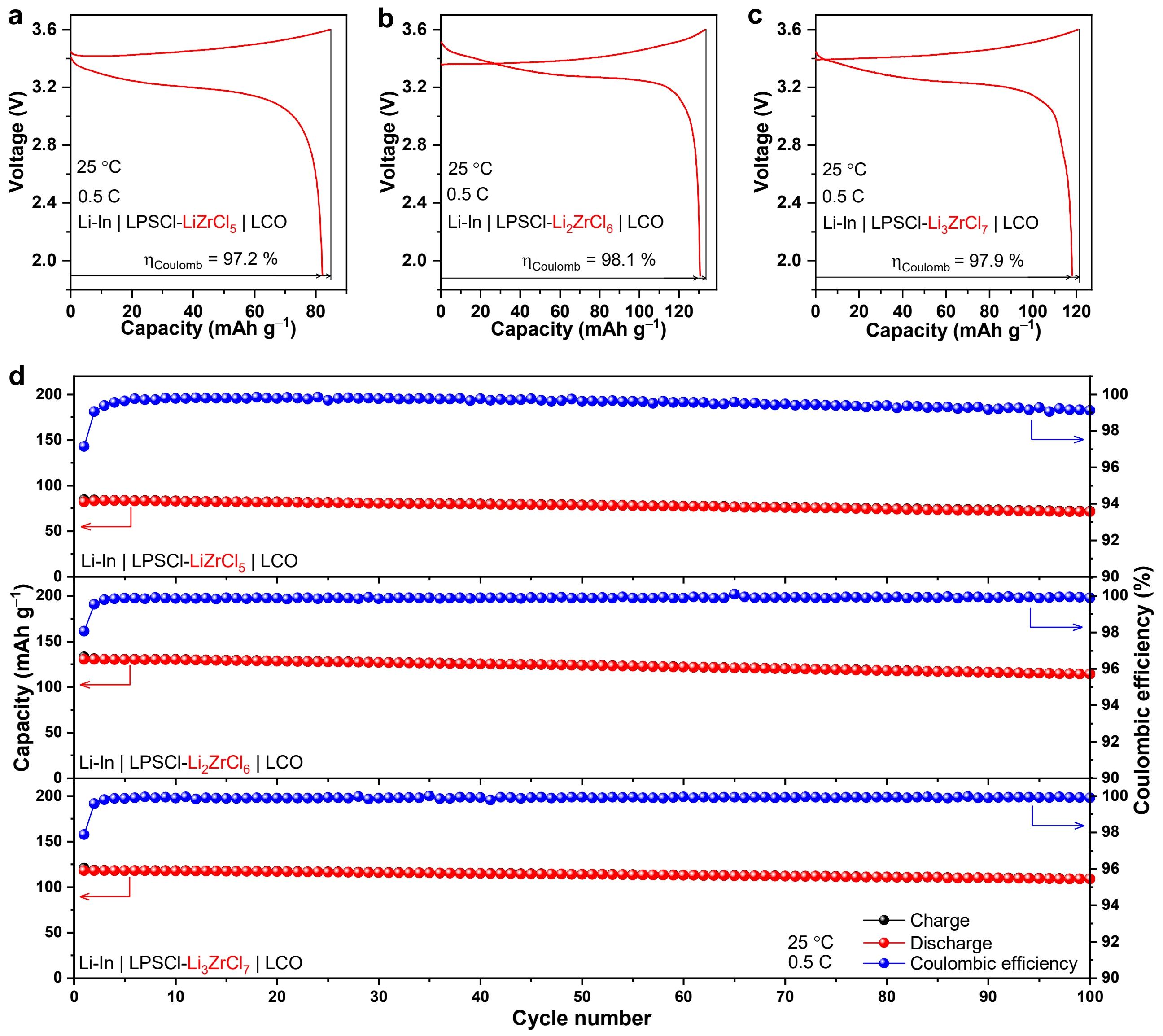

Supplementary Fig. 25. a–c, The initial charge/discharge curves of the all-solid-state Li-In | LPSCl-$LiZrCl_5$ | LCO (**a**), Li-In | LPSCl-Li_2ZrCl_6 | LCO (**b**), and Li-In | LPSCl-Li_3ZrCl_7 | LCO cells (**c**), with the Coulombic efficiency $\eta_{Coulomb}$ denoted. **d**, Long-term cycling performance of the all-solid-state Li-In | LPSCl-$LiZrCl_5$ | LCO, Li-In | LPSCl-Li_2ZrCl_6 | LCO and Li-In | LPSCl-Li_3ZrCl_7 | LCO cells at 0.5 C.

Supplementary Table 1. Comparison of the prices for Li-containing raw materials needed to synthesize different types of solid electrolytes. The bulk prices presented here are estimated from the laboratory-scale prices listed in Supplementary Table 2.

Type of solid electrolyte	Li-containing raw materials	Intercept [$\log_{10}(a)$]	Slope [b]	Absolute correl. coeff. $\|r\|$	Unit price in bulk (1000 kg) purchase, $/kg
Sulfide	Li_2S	4.4081	–0.2654	0.9982	654.18
Oxide	Li_2O	3.8711	–0.0941	0.9639	2025.35
	$LiNO_3 \cdot xH_2O$	4.0134	–0.2189	1	501.19
	$LiNO_3$	2.6010	–0.0789	0.9971	134.15
	Li_2CO_3	2.5498	–0.1296	0.9949	59.18
	LiOH	3.3689	–0.3328	0.9998	23.56
	$LiOH \cdot H_2O$	2.9831	–0.3254	0.9554	10.73
Chloride	LiCl	3.4088	–0.4399	0.9921	5.88

Supplementary Table 2. Laboratory-scale prices used for the estimation in Supplementary Table 1.

All the prices listed here are taken from Alfa Aesar.

Chemical name and description	Stock No.	Purchase quantity, *Q*, grams	Total purchase price, $	Unit price, *P*, $/kg
Lithium sulfide, 99.9% (metals basis)	12839-04	2	43.7	21850
	12839-09	10	136	13600
	12839-18	50	437	8740
	12839-30	250	1524	6096
Lithium oxide, 99.5% (metals basis)	41832-09	10	61.3	6130
	41832-18	50	245	4900
	41832-30	250	1132	4528
Lithium nitrate hydrate, Puratronic®, 99.999% (metals basis)	10742-09	10	62.3	6230
	10742-18	50	219	4380
Lithium nitrate, anhydrous, 99%	13405-30	250	64.9	259.6
	13405-A1	1000	229	229
	13405-A7	5000	1024	204.8
Lithium carbonate, 99%	13418-36	500	78.3	156.6
	13418-A3	2000	271	135.5
	13418-A9	10000	1064	106.4
Lithium hydroxide, anhydrous, 98%	13407-14	25	19.9	796
	13407-22	100	51.1	511
	13407-36	500	147	294
Lithium hydroxide monohydrate, 98%	A15519-22	100	23.6	236
	A15519-36	500	52.8	105.6
	A15519-0E	2500	207	82.8
Lithium chloride, anhydrous, 98+%	A10531-22	100	35.6	356
	A10531-36	500	75.1	150.2
	A10531-0E	2500	216	86.4

Supplementary Table 3. Estimation of the bulk price for different non-Li-containing chlorides based on the laboratory-scale prices listed in Supplementary Table 4.

Chemical formula	Intercept [$\log_{10}(a)$]	Slope [b]	Absolute correl. coeff. $\|r\|$	Unit price in bulk (1000 kg) purchase, \$/kg
$LuCl_3$	5.0899	–0.1055	1	28635.19
$TbCl_3$	4.7716	–0.0561	1	27227.01
$ScCl_3$	5.2533	–0.1687	0.9997	17422.08
$TmCl_3$	5.0142	–0.1452	1	13899.53
$DyCl_3$	4.6872	–0.1218	1	9044.82
$YbCl_3$	4.3940	–0.1027	1	5995.15
$HoCl_3$	4.3143	–0.0907	1	5889.79
$ErCl_3$	3.8968	–0.0934	0.9870	2169.70
$InCl_3$	4.1195	–0.2241	1	595.53
YCl_3	4.0650	–0.2599	0.9967	320.33
$ZrCl_4$	3.3266	–0.3715	1	12.52

Supplementary Table 4. Laboratory-scale prices used for the estimation in Supplementary Table 3. The price for thulium(III) chloride is taken from Fisher Scientific, while those for the other chlorides are from Alfa Aesar.

Chemical name and description	Stock No.	Purchase quantity, *Q*, grams	Total purchase price, $	Unit price, *P*, $/kg
Lutetium(III) chloride, anhydrous, 99.9% (REO)	18685-03	1	123	123000
	18685-06	5	519	103800
Terbium(III) chloride, anhydrous, 99.9% (REO)	41678-03	1	59.1	59100
	41678-06	5	270	54000
Scandium(III) chloride, anhydrous, 99.9% (REO)	18677-01	0.25	56.8	227200
	18677-03	1	178	178000
	18677-06	5	685	137000
Thulium(III) chloride, anhydrous (99.9%-Tm) (REO)	50-901-16191	1	103.32	103320
	50-901-16192	5	408.98	81796
Dysprosium(III) chloride, ultra dry, 99.98% (REO)	35692-06	5	200	40000
	35692-14	25	822	32880
Ytterbium(III) chloride, ultra dry, 99.99% (REO)	40653-06	5	105	21000
	40653-14	25	445	17800
Holmium(III) chloride, anhydrous, 99.9% (metals basis)	18681-06	5	89.1	17820
	18681-14	25	385	15400
Erbium(III) chloride, anhydrous, 99.9% (metals basis)	89917-09	10	64.5	6450
	89917-18	50	266	5320
	89917-30	250	1194	4776
Indium(III) chloride, anhydrous, 98+%	L18758-06	5	45.9	9180
	L18758-14	25	160	6400
Yttrium(III) chloride, anhydrous, 99.9% (REO)	18682-09	10	65.1	6510
	18682-18	50	202	4040
	18682-30	250	705	2820
Zirconium(IV) chloride, 98%, cont. 1-2% hafnium(IV) chloride	L14891-18	50	24.8	496
	L14891-30	250	68.2	272.8

Supplementary Table 5. Estimation of the bulk price for different chloride hydrates from the laboratory-scale prices listed in Supplementary Table 6.

Chemical formula	Intercept [$\log_{10}(a)$]	Slope [b]	Absolute correl. coeff. $\|r\|$	Unit price in bulk (1000 kg) purchase, $/kg
$LuCl_3 \cdot 6H_2O$	4.8075	–0.2376	1	2409.35
$TbCl_3 \cdot 6H_2O$	4.0904	–0.1247	1	2198.87
$ScCl_3 \cdot 6H_2O$	4.9004	–0.2312	1	3259.87
$TmCl_3 \cdot xH_2O$	4.6466	–0.1753	1	3933.69
$DyCl_3 \cdot xH_2O$	3.6562	–0.2234	1	206.92
$YbCl_3 \cdot xH_2O$	3.8042	–0.2066	1	366.94
$HoCl_3 \cdot 6H_2O$	4.0031	–0.2601	1	277.01
$ErCl_3 \cdot xH_2O$	3.4457	–0.0767	1	967.16
$InCl_3 \cdot xH_2O$	4.0581	–0.1742	0.9987	1030.15
$YCl_3 \cdot xH_2O$	3.5339	–0.2768	0.9650	74.66

Supplementary Table 6. Laboratory-scale prices used for the estimation in Supplementary Table 5.

All the prices listed here are taken from Alfa Aesar.

Chemical name and description	Stock No.	Purchase quantity, *Q*, grams	Total purchase price, $	Unit price, *P*, $/kg
Lutetium(III) chloride hexahydrate, REacton®, 99.9% (REO)	11260-03	1	64.2	64200
	11260-06	5	219	43800
Terbium(III) chloride hexahydrate, REacton®, 99.9% (REO)	11209-09	10	92.4	9240
	11209-18	50	378	7560
Scandium(III) chloride hexahydrate, REacton®, 99.9% (REO)	11218-03	1	79.5	79500
	11218-06	5	274	54800
Thulium(III) chloride hydrate, REacton®, 99.9% (REO)	11200-04	2	78.5	39250
	11200-09	10	296	29600
Dysprosium(III) chloride hydrate, 99.9% (REO)	11316-14	25	55.2	2208
	11316-22	100	162	1620
Ytterbium(III) chloride hydrate, REacton®, 99.9% (REO)	11193-09	10	39.6	3960
	11193-18	50	142	2840
Holmium(III) chloride hexahydrate, REacton®, 99.9% (REO)	11277-14	25	109	4360
	11277-22	100	304	3040
Erbium(III) chloride hydrate, REacton®, 99.9% (REO)	11304-14	25	54.5	2180
	11304-22	100	196	1960
Indium(III) chloride hydrate, 99.99% (metals basis)	11859-09	10	75.9	7590
	11859-18	50	294	5880
	11859-30	250	1083	4332
Yttrium(III) chloride hydrate, REacton®, 99.9% (REO)	11184-14	25	37.6	1504
	11184-22	100	83.9	839
	11184-36	500	325	650

Supplementary Table 7. Rietveld refinement result from the room-temperature NPD data of the as-milled LZC. The space group is $P\bar{3}m1$. The refined lattice parameters are a = 10.971(5) Å and c = 5.9309(12) Å.

Atoms	x	y	z	Occ.	site	Sym.	U_{iso} (Å^2)
Li1	0.31(6)	0	0	0.22(17)	6*g*	2	0.034(31)
Li2	0.322(13)	0	1/2	0.78(17)	6*h*	2	0.034(31)
Zr1	0	0	0	0.65(8)	1*a*	-3m	0.005(9)
Zr2	1/3	2/3	0.522(22)	0.57(6)	2*d*	3m	0.005(9)
Zr3	0	0	1/2	0.71(8)	1*b*	-3m	0.005(9)
Zr4	1/3	2/3	0.94(4)	0.25(5)	2*d*	3m	0.005(9)
Cl1	0.1067(6)	-0.1067(6)	0.7521(25)	1	6*i*	m	0.0157(6)
Cl2	0.2281(7)	-0.2281(7)	0.2813(15)	1	6*i*	m	0.0157(6)
Cl3	0.4412(7)	-0.4412(7)	0.7676(15)	1	6*i*	m	0.0157(6)

Supplementary Table 8. Rietveld refinement result from the room-temperature XRD data of the 350 °C-annealed LZC; the initial structure used for the refinement is the model refined from the NPD data of the as-milled LZC at 427 °C. The space group is *C2/m*. The refined lattice parameters are *a* = 6.414(22) Å, *b* = 10.993(11) Å, *c* = 6.422(21) Å, and β = 109.69(5)˚.

Atoms	x	y	z	Occ.	site	Sym.	U_{iso} (Å^2)
Li1	0	0.1495(9)	1/2	0.5	4*h*	2	0.160(22)
Li2	1/2	0	1/2	1	2*d*	2/m	0.160(22)
Zr1	0	0	0	0.27(5)	2*a*	2/m	0.031(5)
Zr2	0	1/3	0	0.364(26)	4*g*	2	0.031(5)
Cl1	0.1905(22)	0.1705(17)	0.2384(16)	1	8*j*	1	0.0127(27)
Cl2	0.2374(29)	0	0.7589(29)	1	4*i*	m	0.0127(27)
